# An information-theoretic proof of the Planckian bound for thermalization

**DOI:** 10.1038/s41567-026-03397-y

**Published:** 2026-09-24

**Authors:** Paolo Abiuso, Alberto Rolandi, John Calsamiglia, Pavel Sekatski, Martí Perarnau-Llobet

**Affiliations:** 1 Institute for Quantum Optics and Quantum Information - IQOQI Vienna, Austrian Academy of Sciences, Vienna, Austria; 2 Atominstitut, TU Wien, Vienna, Austria; 3 Física Teòrica: Informació i Fenòmens Quàntics, Department de Física, Universitat Autònoma de Barcelona, Barcelona, Spain; 4 Département de Physique Appliquée, Université de Genève, Geneva, Switzerland

**Keywords:** Quantum mechanics, Thermodynamics, Quantum information, Quantum metrology

## Abstract

It has been conjectured that the speed of thermalization is universally constrained by a Planckian timescale, *τ*_Pl_ = *ℏ*/(*k*_B_*T*), determined solely by the reduced Planck constant ℏ, Boltzmann’s constant (*k*_B_) and the temperature (*T*). However, a general and model-independent derivation of this limit has remained elusive. Here we provide a general proof of the emergence of Planckian thermalization based on quantum information geometry and quantum metrology. We formulate thermalization as a process that prepares states close to the corresponding thermal ensemble for a set of distinct Hamiltonians, and show that quantum mechanics imposes a universal lower bound *τ* ≥ *τ*_Pl_/2 on the thermalization time *τ* at finite temperature. In the low-temperature regime, the spectral gap *Δ* between the ground and first excited states replaces the temperature as the relevant energy scale and determines the lower bound on the thermalization time, in close connection with the quantum adiabatic theorem. These bounds, rooted in Hamiltonian estimation, establish operational limits on thermalization governed exclusively by fundamental constants and the intrinsic energy scale.

## Abstract (web summary)

Studies of many-body physics have indicated that thermalization processes are bounded by a timescale determined by Planck’s constant. Such a bound has now been proved to apply in general using techniques from quantum information theory.

## Main

Understanding equilibration and thermalization from the underlying reversible quantum dynamics is one of the most fundamental, long-lasting open questions in physics^1–4^. One central aspect of thermalization in quantum many-body systems is the emergence of the Planckian dissipation time *τ*_Pl_ (refs. ^5,6^), as given by:

1 $${\tau }_{{\rm{Pl}}}=\frac{\hslash }{{k}_{{\rm{B}}}T}.$$

This universal timescale grows inversely with temperature *T* and is determined by two fundamental constants in nature, namely the reduced Planck constant *ℏ* and the Boltzmann constant *k*_B_. Analogously to the ‘Planck time’ in quantum gravity, it is conjectured to represent the shortest timescale for thermalization. That is, the minimum time required to reach a Gibbs state starting from an arbitrary non-equilibrium distribution^5,7–11^.

Originally, the concept of a ‘Planck scale of dissipation’, as given by equation (1), was coined by Zaanen to describe the universal behaviour of the conductance of superconductors above their critical temperature^12,13^. This behaviour was comprehensively empirically corroborated in ref. ^14^, which ignited the quest for an underlying microscopic explanation, tentatively put forwards for metals in refs. ^15,16^ and more recently in ref. ^17^, where a bound is provided for the emergence of hydrodynamic behaviour in many-body systems. In parallel, the concept of Planckian time has been linked to the rate of growth of chaos in many-body systems and field theories^18–22^.

Despite the omnipresence of the Planckian time as a fundamental limit on the speed of dissipative or chaotic processes^5,10^, it is not difficult to come up with apparent violations of this conjecture. For instance, in collisional models, thermalization is described by letting the system interact with a bath made up of identical copies of the system in thermal equilibrium^23^. By letting these collisions take place arbitrarily fast, one realizes there is no fundamental restriction on the time required to relax to the thermal state of a previously known Hamiltonian. The best one can hope for, via quantum speed limits, are bounds assuming a limited interaction strength^24–26^.

Motivated by this tension, in this work we propose an approach to rigorously prove the appearance of the Planckian bound for thermalization based on quantum information geometry and metrology. Unlike previous approaches, our assumptions on the structure of the quantum system and the allowed dynamics are minimal and model-independent. Our starting point is to introduce the concept of a thermalization machine *M*, namely a device that thermalizes a quantum system *S*. We make the following basic assumptions on the action of the machine:

- Requirement 1: validity of quantum mechanics. The microscopic evolution of *S* and *M* (including the environment for open systems) is well described by the Schrödinger equation.
- Requirement 2: thermalization. *M* should output states that are close to the thermal ensemble, independently of the specific structure of *S*.

The independence on the exact specifics of *S* in Requirement 2 is crucial and physically motivated, as it makes a clear distinction between state preparation and (chaotic) thermalization processes. In this framework, standard thermal baths satisfying detailed balance^27^ and complex many-body system satisfying the eigenstate thermalization hypothesis^28–30^ would be considered thermalizing machines. Yet our framework is more general, and also covers highly engineered processes, such as algorithms for many-body quantum cooling.

Under these two minimal assumptions, we provide a universal bound (equation (6)) on the time *τ* required to reach thermalization at inverse temperature *β* = 1/*k*_B_*T*. When considering thermalization in a system with a spectral gap Δ—defined as the energy difference between the ground state and the first excited state—our bound can be summarized as follows:

2 $$\tau \ge \left\{\begin{array}{ll}{\tau }_{{\rm{Pl}}}/2,&\beta \varDelta \lesssim 1\\ \hslash /\varDelta ,&\beta \varDelta \gg 1.\end{array}\right.$$

This is in good agreement with previous results in the literature, as Planckian dissipation behaviour is known to break down close to zero temperature^5^.

## An information-theoretic notion of thermalization

Inspired by quantum information theory, our framework is adversarial: it assumes the existence of an entity (the machine *M*) that has full control to realize a desired task (thermalization) in the shortest possible time *τ* under minimal physical constraints. To minimize *τ*, we allow any control on *M* and its interaction with *S*, although they should be independent of the specifics of *S* alone. This is formalized through two main requirements on the thermalizing machine.

### Requirement 1 (validity of quantum mechanics)

*The evolution of* *S* *and* *M* *is described by quantum mechanics*:

3 $$\begin{array}{l} {\dot{\rho }}_{SM}(t)=-\frac{i}{\hslash }[{H}_{S}+{V}_{SM}(t)+{H}_{M}(t),{\rho }_{SM}(t)]\\ {\rho }_{S}(t,{H}_{S}):={{\rm{tr}}}_{M}{\rho }_{SM}(t)\end{array}$$

*where* *H*_*S*_ *is the system Hamiltonian and* *H*_*M*_(*t*) (*V*_*SM*_(*t*)) *is the machine* (*interaction*) *Hamiltonian that specifies the machine and where* ${\rho}_{SM}$ *is the state of the system and machine combined, t the time and* ${{\rm{tr}}}_{M}$*the partial trace on M*.

**Fig. 1:**
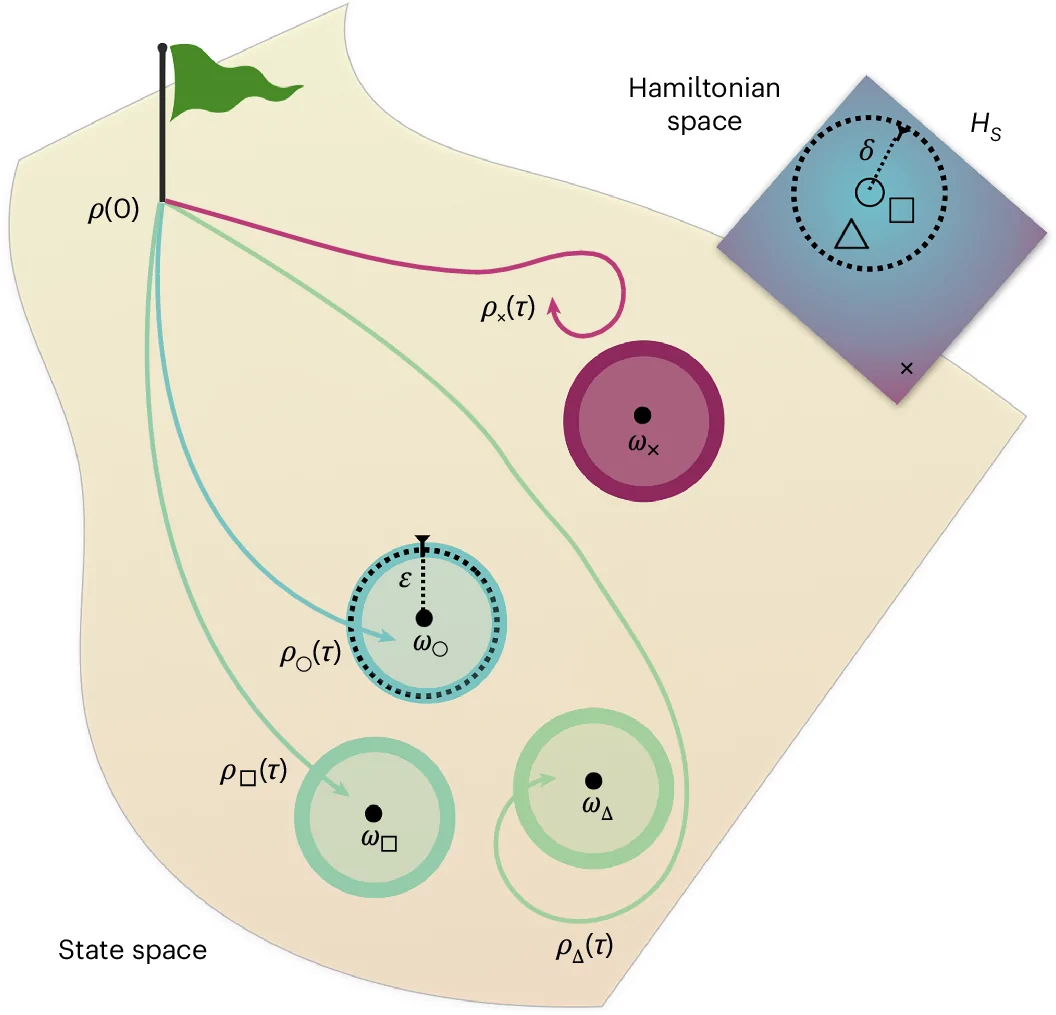
Representation of thermalization process for a given machine *M*. Depending on *H*_*S*_ (in the figure, *H*_*S*_ = ○,△,□,⨯), the dynamical evolution induced by *M* on the initial state of the system *ρ*(0) is required to bring *S* close to the corresponding thermal state *ω*, after time *τ*. This should happen up to an error *ε* and for multiple choices of *H*_*S*_ (here forming a ball of size *δ* for simplicity).

Note that for open quantum systems we include all of the environment in the machine; in other words, we consider any time-dependent unitary dilation of the dynamics. We also emphasize that there is no restriction on the size of *M* and the strengths of *H*_*M*_ and *V*_*SM*_; that is, the machine is free to do anything allowed by quantum mechanics. To represent a non-trivial thermalization process, such a machine should thermalize *S* for multiple choices of *H*_*S*_ via a fixed routine {*H*_*M*_(*t*), *V*_*SM*_(*t*)}. This is illustrated in Fig. 1 and formalized as follows:

### Requirement 2 (thermalization)

*For some* *initial state* *ρ*_*SM*_(0) *and after time* *τ*, *the machine outputs states* *ρ*_*S*_(*τ*, *H*_*S*_) *that are close to the corresponding thermal ensemble at temperature T* = (*k*_B_*β*)^−1^, *for all choices of* *H*_*S*_ *from some set*. *Specifically*, *for a ball* ${{\mathcal{B}}}_{\delta }$ *around* ${\bar{H}}_{S}$ *we require*:

4 $$(i)\quad D\left({\rho }_{S}(\tau ,{H}_{S}),\omega (\beta ,{H}_{S})\right)\le \varepsilon$$

5 $$(ii)\quad \forall \,{H}_{S}\in {{\mathcal{B}}}_{\delta}\,\mathrm{with}\,{{\mathcal{B}}}_{\delta }:=\{H\ \vert \parallel\! H-{\bar{H}}_{S}\!\parallel \le \delta \}\ ,$$

*where* $$\omega (\beta ,{H}_{S}):={\mathrm{e}}^{-\beta {H}_{S}}/{{\mathrm{tr}}}{\mathrm{e}}^{-\beta {H}_{S}}$$ *are Gibbs states*, $$D(\rho ,\sigma ):=\arccos$$$$(\sqrt{F(\rho ,\sigma )})$$ *is chosen to be the Bures angle* (*F* *is the quantum fidelity*) *and* $$\parallel A\!\parallel :={\lambda }_{\max }(A)-{\lambda }_{\min }(A)$$ *is the spectral semi-norm* (*see the* Supplementary Information*for details*). *The parameters* *δ* *and* *ε* *control the range and accuracy of the thermalization machine*, *respectively*.

We claim that requirements 1 and 2 are minimal criteria that should be satisfied to prove the existence of a minimal thermalization timescale. (1) Abandoning Requirement 1 and assuming the validity of classical mechanics at all scales (*ℏ* → 0), would take the Planckian time to zero. (2) Requirement 2 is essential to distinguish a thermalization machine from a single-state preparation machine. Indeed, a collisional ‘swap machine’ can prepare a fixed $\omega (\beta ,{\bar{H}}_{S})$ instantaneously, as discussed above. Finally, it is worth mentioning that both equations (4) and (5) in Requirement 2 are chosen for simplicity and can be relaxed. In particular, for the derivation of our results it is in general sufficient to require an even weaker notion of thermalization, in which equation (4) is satisfied for at least two different *H*_*S*_. Furthermore, it is also sufficient to require equation (4) to be valid for some weighted-average of *ρ*(*t*, *H*_*S*_) with 0 ≤ *t* ≤ *τ* (this includes the possibility of a finite time resolution of the devices) or only after dephasing; for example, in the local energy eigenbasis of *S*. For a detailed discussion see the Supplementary Information.

## The Planckian bound on thermalization

We demonstrate the following lower bound on the thermalization time.

### Result

For any thermalization machine satisfying requirements 1 and 2 the thermalization time must satisfy:

6 $$\begin{array}{rcl}&&\tau \ge {\tau }_{{\rm{Pl}}}\space \chi ({\bar{H}}_{S},\delta ,\varepsilon )\ ,\quad \quad \,\mathrm{with}\,\\ &&\chi ({\bar{H}}_{S},\delta ,\varepsilon ):={\mathop{\max }\limits_{{H}_{S}^{(1,2)}\in {{\mathcal{B}}}_{\delta }}}\left[\frac{2D\left(\omega (\beta ,{H}_{S}^{(1)}),\omega (\beta ,{H}_{S}^{(2)})\right)-4\varepsilon }{\beta \parallel {H}_{S}^{(1)}-{H}_{S}^{(2)}\parallel }\right]\end{array}.$$

This result can be understood as a universal bound on the thermalization time. As we show below, the adimensional factor $\chi ({\bar{H}}_{S},\delta ,\varepsilon )$ in equation (6) is finite and remains bounded from zero in all regimes where thermalization (Requirement 2) remains sufficiently different from single-state preparation. Also note the upper bound $\chi (\bar{H},\delta ,\varepsilon )\le \frac{1}{2}-\frac{4\varepsilon }{\beta \delta }$, which highlights that the accuracy and range parameters must satisfy $\varepsilon \le \frac{\beta \delta }{8}$ to yield a non-trivial bound (in other words, the tolerated error should be sufficiently small to distinguish the different thermal states required).

### Proof sketch

The core idea of the proof is that the distinguishability between the outputs of the thermalization machine generated by different *H*_*S*_ is fundamentally constrained by the sensitivity of the global unitary evolution to changes in *H*_*S*_ (equation (3)). This can be concretized in information-geometrical arguments (see the Supplementary Information for full details): first, when Requirement 2 holds for two Hamiltonians ${H}_{S}^{(1)}$ and ${H}_{S}^{(2)}$, the triangle inequality for the Bures angle implies that $D({\rho }_{S}(\tau ,{H}_{S}^{(1)}),{\rho }_{S}(\tau ,{H}_{S}^{(2)}))\ge D(\omega (\beta ,{H}_{S}^{(1)}),\omega (\beta ,{H}_{S}^{(2)}))-2\varepsilon$. In turn, Requirement 1 sets a limit on variations of *S* under variations of the local *H*_*S*_—after time *τ* the possible states of the system must satisfy $D({\rho }_{S}(\tau ,{H}_{S}^{(1)}),{\rho }_{S}(\tau ,{H}_{S}^{(2)}))\le \frac{\tau }{2\hslash }\parallel\! {H}_{S}^{(1)}-{H}_{S}^{(2)}\!\parallel$. The result (equation (6)) is then obtained by optimizing the choice of the Hamiltonians.

In what follows, we will characterize $\chi ({\bar{H}}_{S},\delta ,\varepsilon )$ in different physically relevant limits to obtain universal bounds on thermalization, and later contrast such bounds with explicit dynamics/machines.

#### Locally exact thermalization and quantum Fisher information

Let us now consider the case of locally exact thermalization, by making the ball of Hamiltonians *δ* → 0 in Requirement 2 infinitesimal, while keeping the error on the thermal state *ε* ≪ *βδ* negligible. In this limit, the machine must prepare the exact Gibbs state *ρ*_*S*_(*τ*, *H*_*S*_) ≡ *ω*(*β*, *H*_*S*_), for all perturbations of the Hamiltonian ${H}_{S}(\delta ,\kappa )={\bar{H}}_{S}+\delta \kappa$ with *κ* any hermitian operator satisfying ∥ *κ* ∥ ≤ 1 and infinitesimal *δ*. The bound (equation (6)) then becomes:

7 $$\tilde{\chi }({\bar{H}}_{S}):={\mathop{\lim }\limits_{\frac{\varepsilon }{\beta }\ll \delta \to 0}}\chi ({\bar{H}}_{S},\delta ,\varepsilon )={\mathop{\max }\limits_{\kappa ={\kappa }^{\dagger }}}\frac{\sqrt{{{\mathcal{F}}}_{\kappa }^{{\rm{th}}}(\beta ,{\bar{H}}_{S})}}{\beta \parallel \kappa \parallel },$$

where ${{\mathcal{F}}}_{\kappa }^{{\rm{th}}}(\beta ,{\bar{H}}_{S})\equiv {\mathcal{F}}\left(\omega (\beta ,{H}_{S}(0,\kappa ))\right)$ is the quantum Fisher information (QFI) of a thermal state^31–33^. Here we used the fact that the QFI of a parametric state *ρ*_*δ*_ is related to its susceptiblity with respect to the Bures angle via ${\mathcal{F}}({\rho }_{0})=4{({\lim }_{\delta \to 0}\frac{D({\rho }_{0},{\rho }_{\delta })}{\delta })}^{2}$ (Supplementary Information).

This result admits a natural interpretation in terms of quantum metrology. For locally exact thermalization, ${{\mathcal{F}}}_{\kappa }^{{\rm{th}}}(\beta ,{\bar{H}}_{S})$ must coincide with the ‘dynamical’ QFI ${{\mathcal{F}}}_{\kappa }^{{\rm{dyn}}}(\tau ,{\bar{H}}_{S})\equiv {\mathcal{F}}\left({\rho }_{S}(\tau ,{H}_{S}(0,\kappa ))\right)$, which is bounded by the generalized Heisenberg limit ${{\mathcal{F}}}_{\kappa }^{{\rm{dyn}}}(\tau ,{\bar{H}}_{S})\le\parallel\kappa \parallel ^{2}{\tau }^{2}/{\hslash }^{2}$ (ref. ^34^). By maximizing over the Hamiltonian perturbations *κ*, we then immediately recover equation (7). In other words, this bound follows from the observation that the thermalization machine cannot violate the Heisenberg limit.

The maximization in equation (7) is detailed in the Supplementary Information, where we show that $\tilde{\chi }\ge \sqrt{p(1-p)}$ for all possible bipartitions of the population of $\omega (\beta ,{\bar{H}}_{S})$ in two sets with probabilities {*p*, 1 − *p*}. When $\omega (\beta ,{\bar{H}}_{S})$ is sufficiently mixed that *p* ≈ 1/2 can be chosen, we find $\tilde{\chi }\approx 1/2$, recovering the first line of equation (2). In particular, $\tilde{\chi }\ge \sqrt{2}/3 \approx 0.47$ whenever the ground-state probability *p*_0_ is below 2/3. For the case *p* → 1, namely as $\omega (\beta ,{\bar{H}}_{S})$ approaches the ground state, we derive a different bound that is tighter in such a regime: $\tilde{\chi }\ge (2{p}_{0}-1)/(\beta \varDelta )$ for *β*Δ ≫ 1. This leads to the second line of equation (2).

#### Approximate thermalization

The limit of locally exact thermalization yields simple bounds and an intuitive understanding in terms of the Fisher information. The more general inequality (6) follows from a similar geometrical argument using finite variations of *H*_*S*_ while admitting the possibility of a finite error *ε* > 0 in reaching the exact thermal state. Such a possibility is crucial to ensure the continuity and, more importantly, the wide validity of our main results: thermalization in nature is not, in general, exact.

**Fig. 2:**
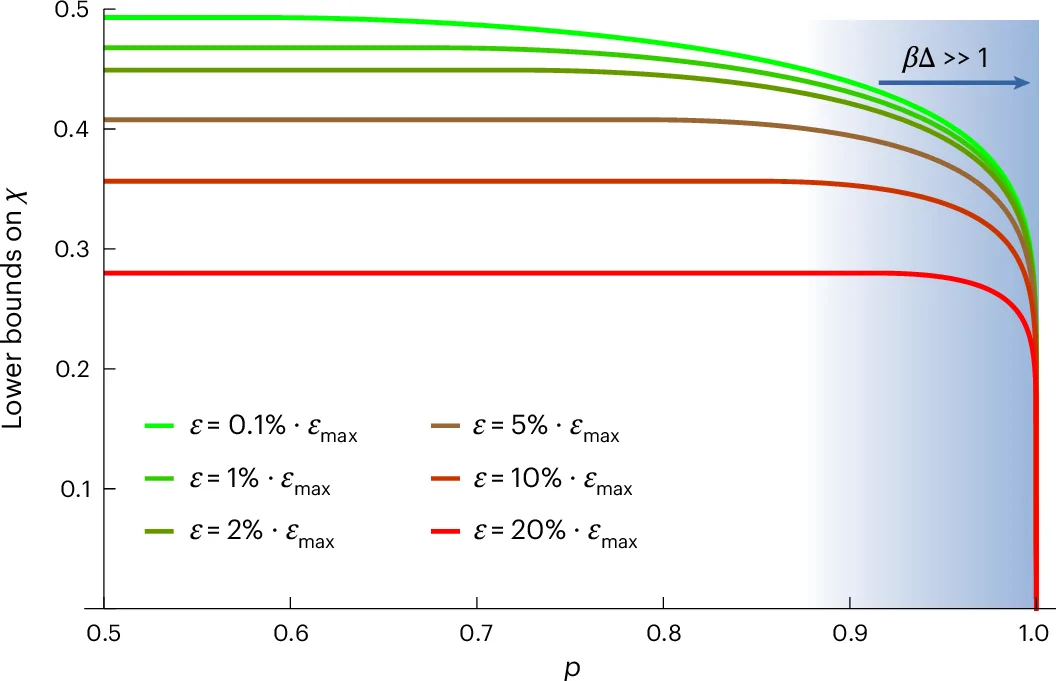
Bounds for approximate thermalization. For different finite values of *ε*, lower bounds on $\chi ({\bar{H}}_{S},\delta ,\varepsilon )$ are shown as a function of any bipartition {*p*, 1 − *p*} of the thermal state populations at ${\bar{H}}_{S}$. Specifically, equation (6) is partially optimized over a simple set of Hamiltonians ${H}_{S}^{(i)}$, namely those that commute with ${\bar{H}}_{S}$ and for which ${H}_{S}^{(i)}-{\bar{H}}_{S}$ has only two distinct eigenvalues. In units of Bures angle, the maximum tolerated error corresponds to *ε*_max_ ≡ π/4. For *ε* ≲ 5% ⋅ ε_max_, *χ* is in general at least greater than ~0.4. In the limit *p* → 1 (that is, close to the ground state), we find that *χ* tends to zero slower than 1/(*βΔ*) (see the Supplementary Information for details).

On a formal level, moving away from *ε* = 0 makes the function $\chi ({\bar{H}}_{S},\delta ,\varepsilon )$ (equation (6)) more challenging to compute. However, we can compute again different lower bounds that hold for any ${\bar{H}}_{S}$ in any Hilbert space dimension and depend only on the possible bipartite coarse-graining of the thermal state populations. These bounds are shown in Fig. 2 and we observe once again how they yield *χ* ≳ 0.5 for states that are sufficiently mixed (that is, when $\omega (\beta ,{\bar{H}}_{S})$ is not concentrated only in the ground state) and sufficiently small errors. As *ε* increases, the machine might in principle become faster; however, note that at ~5% error one still has *χ* ≳ 0.4 and at around 20% error, *χ* ≳ 0.3. Remarkably, all lower bounds in Fig. 2 can be obtained by considering a simple subset of Hamiltonians that are diagonal in the basis of ${\bar{H}}_{S}$. That is, they hold even when *M* is required to thermalize only classical (commuting) Hamiltonians.

#### Applications to model-informed scenarios

After deriving model-independent limits on the preparation of thermal states, we now outline how the techniques that we introduced can be applied well beyond this scenario and how refined bounds can be derived when: (1) only specific observables can be measured; (2) the required outputs are not necessarily thermal; or (3) further details of the physical systems involved are given.

For simplicity, we take the limit of locally exact functioning of *M* (that is, negligible *ε* and infinitesimal *δ*), in that the machine is only required to operate close to ${H}_{S}={\bar{H}}_{S}+\delta \kappa$. Suppose that the user of the machine does not want to retrieve thermal states *ω*(*β*, *H*_*S*_) exactly, but rather a generic state $\tilde{\omega }({H}_{S})$ that has a dependence on its Hamiltonian (this includes reduced Gibbs states, generalized Gibbs ensembles, dephased states, steady states of noisy systems and so on). Second, the user is not able to obtain full tomography of the output: rather, they can only measure some observable *A* = ∑_*a*_*aΠ*_*a*_, *Π*_*a*_ being the projector corresponding to output *a*. Then, the accessible statistics of the user are locally limited to ${p}_{a}={\rm{tr}}{\varPi }_{a}\tilde{\omega }({\bar{H}}_{S}+\delta \kappa )$, with *δ*-derivative ∂*p*_*a*_ at ${\bar{H}}_{S}$. By definition, the corresponding accessible Fisher information is upper-bounded by the QFI of $\tilde{\omega }$: ${\sum }_{a}\frac{{(\partial {p}_{a})}^{2}}{{p}_{a}}\le {{\mathcal{F}}}_{\kappa }^{\tilde{\omega }}$. After forcing the latter to be equal to the dynamical QFI ${{\mathcal{F}}}_{\kappa }^{{\rm{dyn}}}{| }_{S}$ on *S*, and noticing that this is smaller than the global QFI of the unitarily evolving *S* + *M*, one can derive a refined bound via convexity (Supplementary Information):

8 $$\sum _{a}\frac{{(\partial {p}_{a})}^{2}}{{p}_{a}}\le {{\mathcal{F}}}_{\kappa }^{\tilde{\omega }}\le {{\mathcal{F}}}_{\kappa }^{{\rm{dyn}}}{| }_{SM}\le \frac{{\tau }^{2}}{{\hslash }^{2}}\int_{0}^{1}{\rm{d}}s\ {{\mathcal{F}}}_{\kappa }^{{\rm{U}}}({\rho }_{SM}(s\tau ))\ ,$$

where ${{\mathcal{F}}}_{\kappa }^{{\rm{U}}}(\rho )$ is the Fisher information obtained by a unitary rotation of state *ρ* with generator *κ*. In the absence of further knowledge on *M* the latter is upper-bounded by ${{\mathcal{F}}}_{\kappa }^{{\rm{U}}}\le\parallel\kappa \parallel ^{2}$, hence when the user can measure any observable *A* and the required output $\tilde{\omega }$ is thermal, this directly leads to the general bound (7). However, we stress here that equation (8) can be applied to any (model-specific) scenario of interest. Moreover, energy measurements are typically sufficient to saturate the first inequality in equation (8) (Supplementary Information).

As an example, consider the system *S* to be *N*-partite and $\kappa =\mathop{\sum }\nolimits_{i = 1}^{N}{\kappa }^{(i)}$ to be a uniform perturbation—for example, when *δ* is an intensive order parameter of a phase-transition. One can then immediately turn the above inequality in

9 $$\tau \gtrsim \beta \hslash {N}^{\frac{\alpha -\phi }{2}},$$

where *N*^*α*^ represents the scaling of the thermal QFI of *S* (possibly at criticality), and *N*^*ϕ*^ that of the global dynamical QFI of *S* + *M*. Standard uncorrelated thermal systems satisfy *α* = 1, while it has been proved in ref. ^32^ that local classical observables can achieve up to *α* = 2 on strongly correlated thermal systems. Moreover, *ϕ* = 2 needs the machine dynamics to generate consistent *N*-partite entanglement in *ρ*_*SM*_, whereas *ϕ* = 1 when a separable partition can be found at all times (Supplementary Information).

One can also apply equation (8) when further structure of the model is known. Consider a many-body closed system *SM* thermalizing on *S*, under the additional assumptions that the entire *SM* is initially uncorrelated among its constituents and the overall Hamiltonian is local; one can then apply Lieb–Robinson-type bounds to the growth of entanglement in time^35^. In particular, for short-range interactions, these are known to bound ${{\mathcal{F}}}_{\kappa }^{{\rm{U}}}\lesssim N{(\nu t)}^{d}$, where *ν* is the light-cone speed on the many-body lattice and *d* its spatial dimension. It follows then from equation (8) that ${{\mathcal{F}}}_{\kappa }^{{\rm{dyn}}}\lesssim \frac{{\tau }^{2}}{{\hslash }^{2}}N\int_{0}^{1}{(\nu s\tau )}^{d}=\frac{{\tau }^{2}}{{\hslash }^{2}}N\frac{{(\nu \tau )}^{d}}{d+1}$ and therefore:

10 $$\begin{array}{r}{\tau }^{2+d}\gtrsim {\beta }^{2}{\hslash }^{2}{N}^{\alpha -1}\frac{d+1}{{\nu }^{d}}\ .\end{array}$$

As expected, equation (10) is tighter than equation (9) in the range for which the Lieb–Robinson bound is informative 1 < (*ντ*)^*d*^ < *N*^*α*−1^.

## Approaching the Planckian limit

After deriving and analysing the fundamental inequality (equation (6)), we now address its tightness; that is, are there specifically engineered thermalization machines that can approach the Planckian time? As our bound is a consequence of the machine’s inability to thermalize two different Hamiltonians in a given time, a preliminary attempt is offered by Hamiltonian discrimination, where it is known that two Hamiltonians ${H}_{S}^{(1)}$ and ${H}_{S}^{(2)}$ can be perfectly distinguished after time *τ* if, and only if, $\tau \ge \hslash \uppi / \!\parallel \!{H}_{S}^{(1)}-{H}_{S}^{(2)}\!\parallel$ (ref. ^36^). Clearly, this time is also sufficient for thermalization, as preparing the Gibbs state of a known Hamiltonian is trivial in our framework. This ‘discrimination’ time, however, is longer than our lower bound (equation (6)), and is even diverging for nearby Hamiltonians. Inspired by this discrimination task, we now describe a machine that saturates the Planckian bound for any given pair of Hamiltonians.

First, note that after time *τ* the machine used for Hamiltonian discrimination^36^ prepares the system in two pure states |*ψ*_i_(*τ*)〉_*S*_, corresponding to the Hamiltonians $${H}_{S}^{(i)}$$, such that $$| \langle {\psi }_{1}(\tau )| {\psi }_{2}(\tau )\rangle {| }^{2}$$$$={\cos }^{2}(\frac{\tau }{2\hslash }\!\parallel \!{H}_{S}^{(1)}-{H}_{S}^{(2)}\!\parallel)$$ (see the Supplementary Information for details). With an isometry *V* the machine can then map these two states to any pair of pure states $${\left\vert {\varPsi }_{i}\right\rangle }_{SM}=V{\left\vert {\psi }_{i}(\tau )\right\rangle }_{S}$$ that are purifications of *ω*(*β*, *H*^(*i*)^). Using Uhlmann’s theorem, we can choose these states so that they satisfy ∣〈*Ψ*_1_∣*Ψ*_2_〉∣^2^ = *F*(*ω*(*β*, *H*^(1)^), *ω*(*β*, *H*^(2)^)). Hence, our machine can thermalize the two Hamiltonian after time:

11 $$\tau =\frac{2\,\hslash \,D(\omega (\beta ,{H}^{(1)}),\omega (\beta ,{H}^{(2)}))}{\parallel {H}_{S}^{(1)}-{H}_{S}^{(2)}\parallel },$$

saturating the bound (6) for *ε* = 0.

## A case study: resonant-level model of thermalization

**Fig. 3:**
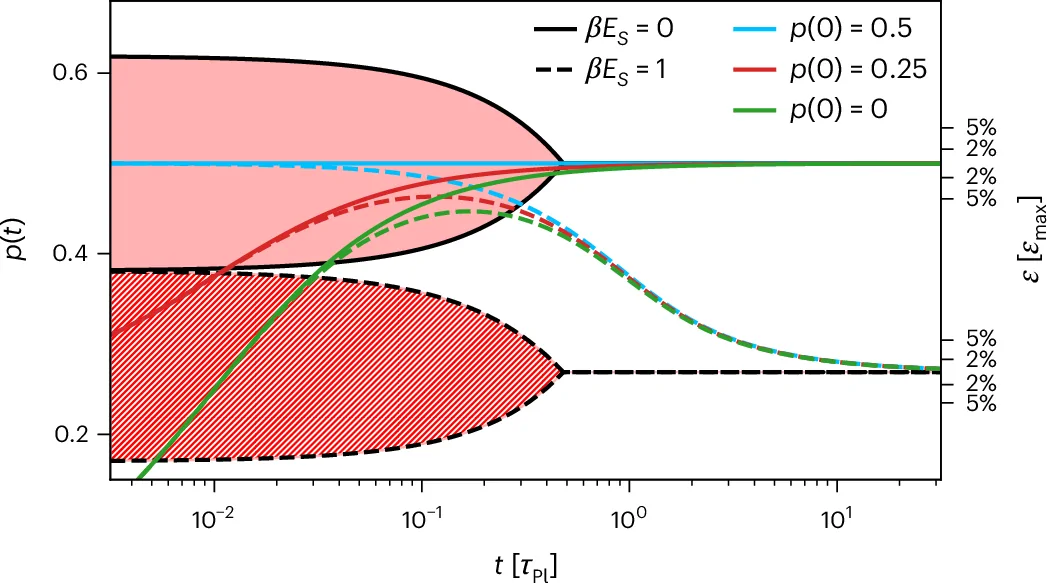
Exact evolution of a fermionic model versus a Planckian thermalization bound. For each value of *ε* our bound (equation (6)) sets a minimum time *τ* under which a machine is unable to thermalize different Hamiltonians simultaneously. For the same initial condition, if a given machine quickly thermalizes one Hamiltonian ${H}_{S}^{(1)}$ (red shaded area and solid lines) then it cannot thermalize a second Hamiltonian ${H}_{S}^{(2)}$ (red hatched area and dashed lines) with arbitrary precision in the same amount of time. This must hold for any conceivable machine *M* that is able to thermalize *S*. Here we illustrate it for the fermionic machine described in the text (on the left vertical axis we plot $p(t):={\rm{tr}}{\rho }_{S}(t){\hat{a}}^{\dagger }\hat{a}$, whereas on the right vertical axis we indicate the Bures angle distance to each corresponding thermal state, in units of the maximum error). As can be seen from the plot, the evolution is such that if one of the two Hamiltonians is thermalized faster than the Planckian time (solid black line in the red shaded area or dashed black line in the red hatched area), then the other is not. Only after a time on the order of the Planckian time has passed can we see that both Hamiltonians can thermalize.

The above example shows how a fine-tuned machine could, in principle, approach the Planckian limit of thermalization. We now discuss a physical model in which *S* is a single fermion described by (commuting) Hamiltonians ${H}_{S}={E}_{S}{\hat{a}}^{\dagger }\hat{a}$, and thermalization can be characterized through its occupation probability $p(t):={\rm{tr}}{\rho }_{S}(t){\hat{a}}^{\dagger }\hat{a}$. Starting from the same initial state, the bound from equation (6) forbids any machine to concurrently thermalize multiple Hamiltonians in less than *t* = *τ*_Pl_ ⋅ *χ*. That is, when the evolution with a Hamiltonian ${H}_{S}^{(1)}$ is too close to the thermal state $\omega (\beta ,{H}_{S}^{(1)})$, then the evolution with another Hamiltonian ${H}_{S}^{(2)}$ has to be far from $\omega (\beta ,{H}_{S}^{(2)})$ and vice versa. In Fig. 3 we visualize this with the red regions by picking two Hamiltonians with energy gap ${E}_{S}^{(1)}=0$ and ${E}_{S}^{(2)}={k}_{\mathrm{B}}T$. At *t* = 0 the size of these forbidden areas (in red) is maximal, and as time increases to the order of the Planckian time, the size decreases to zero; in Fig. 3 the bound becomes trivial at *t* ≈ *τ*_Pl_/2.

As a testing playground, we chose a machine *M* consisting of a fermionic bath with Hamiltonian ${H}_{M}={\sum }_{k}{\varOmega }_{k}{\hat{b}}_{k}^{\dagger }{\hat{b}}_{k}$ of *n* fermionic modes ${\hat{b}}_k$ and energy ${\varOmega }_{k}$ that can interact with *S* via tunnelling interactions ${V}_{SM}(t)=g(t)\mathop{\sum }\nolimits_{k = 1}^{n}{\lambda }_{k}{\hat{a}}^{\dagger }{\hat{b}}_{k}+{\lambda }_{k}^{* }{\hat{b}}_{k}^{\dagger }\hat{a}$, the so-called resonant-level model^37,38^. The time-dependent function *g*(*t*) characterizes the machine’s operating schedule. For initially uncorrelated *S* and *M* we can solve the exact dynamics in the thermodynamic limit (Supplementary Information) for any choice of local energy gap *E*_*S*_ and coupling *g*(*t*). We analysed schedules of the form $g(t)=\sqrt{a/(t+b)}$ for 0 < *t* < *τ*, *a*, *b* > 0 in detail. We observe in Fig. 3 that these trajectories approach the Planckian limit, while satisfying our constraints.

## Discussion

We presented a fundamental time bound (equation (6)) valid for any thermalization process in quantum mechanics. To formalize our results in the most general framework, we introduced the concept of the thermalization machine, *M*, which only has to comply with quantum mechanics and output states close to thermal equilibrium. As such, it applies to any device (including quantum computers) attempting to prepare thermal states for a set of Hamiltonians. This therefore connects two tasks that have been intensively studied in recent years, namely Gibbs state sampling^39–44^ and Hamiltonian learning^45–47^ (Table 1). Our results can be summarized as equation (2): if *M* is required to thermalize all Hamiltonians *H*_*S*_, or at least those leading to thermal states that are sufficiently mixed (*βΔ* ≲ 1), *τ*_Pl_/2 = *ℏ*/(2*k*_B_*T*) sets the universal minimum operational time of *M*. When the the machine is only required to be a ground-state cooling machine, its minimum operational time is bounded by (less stringent) *ℏ*/*Δ*, consistent with optimal protocols for adiabatic state preparation^33,48^. The core idea behind these results lies in comparing the distinguishability of thermal states with the global variation of unitary dynamics induced by their Hamiltonians.

**Table 1:** Information protocols based on Hamiltonian knowledge

	Gibbs state sampling	Thermalization machine	Hamiltonian learning
Input	Exact knowledge of *H*	Oracle access to *H*	Oracle access to *H*
Desiderata	Thermal statistics	Thermal statistics	*H* discrimination
Minimum time (for two values of *H*)	0	*τ*_Pl_/2 (this work)	*τ*_AMP_ (ref. ^36^)

Gibbs state sampling requires reproducing the thermal statistics induced by some Hamiltonian *H* given its classical description. In the case of Hamiltonian learning the goal is more demanding (learning *H*) and the premise is weaker (some kind of oracle access to *H*). In this context, our thermalization machines share the desiderata with GSS, and the input with Hamiltonian learning. In the table, the information provided to the machine decreases from left to right, while the output information required increases. As a consequence, these tasks are increasingly harder, and the fundamental minimum time needed by an all-powerful entity to accomplish these tasks is ordered accordingly; when only two possible values of *H* are given, and the entity is only limited to obey quantum mechanics, GSS can in principle be arbitrarily fast—via a control-swap machine—whereas thermalization and Hamiltonian learning have to comply with the Planckian bound (equation (6)) and the Hamiltonian discrimination bound $\tau_{{\mathrm{AMP}}}:=\hbar\pi/({\parallel \!{H}_{S}^{(1)}-{H}_{S}^{(2)}\!\parallel })$^36^.

While we focused on the ultimate bounds imposed by quantum mechanics alone, our results raise further questions: in multipartite systems, what is the role of entanglement in the Planckian bound? What happens in the large-size limit? How close to the Planckian bound can realistic baths approach? A comprehensive answer to these questions is not within the scope of this work and motivates further investigation, but we showed how our techniques can be used to refine our bound to specific scenarios, once additional details are known about the physical systems involved. Finally, we note that as *τ*_Pl_ ∝ 1/*T*, the bound *τ* ≳ *τ*_Pl_ is, in its range of validity, reminiscent of the third law of thermodynamics, thus providing geometric insight into it.

## Online content

Any methods, additional references, Nature Portfolio reporting summaries, source data, extended data, supplementary information, acknowledgements, peer review information; details of author contributions and competing interests; and statements of data and code availability are available at https://doi.org/10.1038/s41567-026-03397-y.

## Supplementary information

Supplementary Information Supplementary analysis and technical details.

Peer Review File

## Data Availability

Numerical data are available on request from the corresponding authors.
