# Supplementary material for: An information-theoretic proof of the Planckian bound for thermalization
Source: Nat Phys. 2026 Sep 24;22(11):1761–6. doi: 10.1038/s41567-026-03397-y (PMC13759322; doi:10.1038/s41567-026-03397-y)
Supplement: Supplementary file 1 [file 41567_2026_3397_MOESM1_ESM.pdf]

nature physics

Supplementary information

https://doi.org/10.1038/s41567-026-03397-y

An information-theoretic proof of the Planckian bound for thermalization

In the format provided by the authors and unedited

Supplemental Material for "An information-theoretic proof of the Planckian bound for thermalization"

CONTENTS

A. Preamble: quantum operators, Fisher information, notation.
B. Finite distance and QFI for unitary evolutions, generalized Heisenberg limit, entanglement.
C. General framework and Planckian bound derivation.
D. Lowerbounds to $\tilde{\chi}(\rho)$ (locally-exact thermalization).
E. Lower bounds to $\chi(\mathcal{H},\varepsilon)$ (finite-size/finite-error thermalization).
F. The optimal thermalization machine for two Hamiltonians.
G. Resonant level model.

A. PREAMBLE: QUANTUM OPERATORS, FISHER INFORMATION, NOTATION.

In this appendix, we remind the definitions of common mathematical quantities used in the context of quantum information, as well as operators that we use in the derivation of our results.

Fidelity, Bures angle, distances and joint convexity. The fidelity F between two quantum states ρ and σ is given by

$$F(\rho,\sigma) = F(\sigma,\rho) := \mathrm{Tr}\left[\sqrt{\sqrt{\sigma}\rho\sqrt{\sigma}}\right]^2 , \tag{A.1}$$

and it can achieve values between $F = 0$ (perfectly distinguishable, orthogonal states) and $F = 1$ ($\rho = \sigma$). The *Bures angle* (or quantum Bhattacharyya angle) is given by

$$D_A(\rho,\sigma) := \arccos\left(\sqrt{F(\rho,\sigma)}\right) , \tag{A.2}$$

and is bounded as $0 \leq D_A \leq \frac{\pi}{2}$ accordingly. The related *Bures distance* $D_B(\rho,\sigma)$ between two quantum states is defined as

$$D_B(\rho,\sigma)^2 := 2\left(1 - \sqrt{F(\rho,\sigma)}\right) . \tag{A.3}$$

$D = D_B, D_A$ both define a distance on the set of states and satisfy in particular the triangular inequality

$$D(\rho,\sigma) + D(\sigma,\kappa) \geq D(\rho,\kappa) \tag{A.4}$$

which is crucial to our purposes. Moreover both the squared Bures distance and squared Bures angle are additionally *jointly convex*, namely

$$D^2(\lambda\rho_1 + (1-\lambda)\rho_2, \lambda\sigma_1 + (1-\lambda)\sigma_2) \leq \lambda D^2(\rho_1,\sigma_1) + (1-\lambda)D^2(\rho_2,\sigma_2) . \tag{A.5}$$

While this property is known for D_B^2, it is slightly less known for D_A^2, reason for which we shortly prove it here for the reader. First, notice that the joint convexity of D_B^2 is due to the joint concavity property of the square root fidelity, i.e.

$$\sqrt{F(\lambda\rho_1 + (1-\lambda)\rho_2, \lambda\sigma_1 + (1-\lambda)\sigma_2)} \geq \lambda\sqrt{F(\rho_1,\sigma_1)} + (1-\lambda)\sqrt{F(\rho_2,\sigma_2)} . \tag{A.6}$$

Second, notice that the function $\arccos(x)^2$ is convex and decreases monotonically for $0 \leq x \leq 1$. It follows that

$$\begin{aligned}\arccos^2\left(\sqrt{F(\lambda\rho_1 + (1-\lambda\rho_2), \lambda\sigma_1 + (1-\lambda)\sigma_2)}\right) &\leq \arccos^2\left(\lambda\sqrt{F(\rho_1,\sigma_1)} + (1-\lambda)\sqrt{F(\rho_2,\sigma_2)}\right)\\ &\leq \lambda \arccos^2\left(\sqrt{F(\rho_1,\sigma_1)}\right) + (1-\lambda)\arccos^2\left(\sqrt{F(\rho_2,\sigma_2)}\right) ,\end{aligned} \tag{A.7}$$

where the first inequality follows from (A.6) and the decreasing monotonicity of $\arccos^2(x)$, and the second inequality from the convexity of $\arccos^2(x)$. Eq. (A.7) shows the joint convexity of D_A^2.

Bures metrics and Quantum Fisher information. Locally, the Bures angle and Bures distance both define the same Riemannian metric on the set of density matrices[1]

$$D_B(\rho,\rho+\delta\rho)^2 \simeq D_A(\rho,\rho+\delta\rho)^2 \simeq \frac{1}{4}\langle\!\langle\delta\rho|\delta\rho\rangle\!\rangle|_\rho + \mathcal{O}(|\delta\rho|^3) \tag{A.8}$$

that is quantified by the scalar product associated to the (Bures) Quantum Fisher information (QFI), namely

$$\langle\!\langle\delta\rho|\delta\rho\rangle\!\rangle|_\rho = \mathrm{Tr}\left[\delta\rho\mathbb{J}_\rho^{-1}[\delta\rho]\right] \ , \quad \text{where} \quad \mathbb{J}_\rho[A] = \frac{\rho A + A\rho}{2} \ . \tag{A.9}$$

$\mathbb{J}_\rho$ can be inverted as

$$\mathbb{J}_\rho[A] := \frac{\rho A + A\rho}{2} \ , \qquad\qquad \mathbb{J}_\rho^{-1}[A] = 2\int_0^\infty \mathrm{d}s\, e^{-\rho s}Ae^{-\rho s}. \tag{A.10}$$

The $\mathbb{J}$ operator belongs to the family of generalized quantum Petz-Fisher multiplications [33] [i], which represent non-commutative versions of the multiplication times ρ. These operators are all self-adjoint w.r.t. the Hilbert-Schmidt scalar product, meaning that $\mathrm{Tr}\left[A\mathbb{J}[B]\right] = \mathrm{Tr}\left[\mathbb{J}[A]B\right]$ for Hermitian A, B. In addition to their operator-integral expressions above, notice that the action of these superoperators can be explicited in the components $|i\rangle\langle j|$, where $|i\rangle$ are the eigenvectors of ρ. That is, given

$$\rho = \sum_i p_i \,|i\rangle\langle i| \ , \tag{A.11}$$

one has

$$\mathbb{J}_\rho[|i\rangle\langle j|] = \frac{p_i + p_j}{2}\,|i\rangle\langle j| \ . \tag{A.12}$$

In general, the QFI $\mathcal{F}(\rho_\theta)$ of a state parametrized by some θ can be equivalently expressed as

$$\mathcal{F}(\rho_\theta) := \langle\!\langle\partial_\theta\rho_\theta|\partial_\theta\rho_\theta\rangle\!\rangle|_{\rho_\theta} = \mathrm{Tr}\left[\partial_\theta\rho_\theta\mathbb{J}_{\rho_\theta}^{-1}[\partial_\theta\rho_\theta]\right] \equiv \lim_{\delta\theta\to 0} 4\frac{D(\rho_\theta,\rho_{\theta+\delta\theta})^2}{\delta\theta^2} \ . \tag{A.13}$$

NOTE:
in the rest of the supplementary material and throughout the main text,
we simply indicate the Bures angle D_A (A.2) as D.

Spectral seminorm, spectral norm, and thermodynamics. The spectral seminorm $\|\ldots\|$ of a hermitian operator is given by the difference of the maximum and minimum eigenvalues

$$\|A\| := \lambda_{\max}(A) - \lambda_{\min}(A) \ . \tag{A.14}$$

Notice that $\|\ldots\|$ is a seminorm as it satisfies homogeneity $\|\eta A\| = \eta\|A\|$ and the norm inequality $\|A+B\| \le \|A\| + \|B\|$. However it can be zero for nonnull operators, namely those proportional to the identity operator, $\|\eta\mathbb{I}\| \equiv 0$. We can define the equivalence relation between operators $A \sim B$ if $A - B \propto \mathbb{I}$. In such case, the quotient space of operators is still a vector space, with a null vector given by the class $0 := [\mathbb{I}]$. On such space, $\|\ldots\|$ is a proper norm. Notice that the quotient space of Hermitian operators (bounded from below in the infinite-dimensional case) is exactly the natural space of Hamiltonians, as energy is defined up to a constant offset, and H and $H + \eta\mathbb{I}$ define the same thermal state for any η.

---

[1] D_B represents the geodesics distance relative to (A.8) between ρ and σ in the manifold of positive semidefinite matrices, whereas D_A corresponds to the same metrics integrated on the manifold of normalized density matrices satisfying the additional constraint $\mathrm{Tr}\left[\rho\right] = 1$, see e.g. [33].

B. FINITE DISTANCE AND QFI FOR UNITARY EVOLUTIONS, GENERALIZED HEISENBERG LIMIT, ENTANGLEMENT.

Consider the reduced state of a unitary evolution, as in the main text is the system S, that is

$$\rho_S(t, H_S) = \mathrm{Tr}_M \rho_{SM}(t) = \mathrm{Tr}_M\, U(t)\rho_{SM}(0)U^\dagger(t)\ , \tag{B.1}$$

where

$$U(t) = \mathcal{T}\exp\left\{\frac{i}{\hbar}\int_0^t \mathrm{d}s\ H_{tot}(s)\right\}\ ,\quad H_{tot}(s) = H_S + V_{SM}(s) + H_M(s)\ . \tag{B.2}$$

Our goal is to upper bound the value of the finite Bures angle between two states that evolved with different Hamiltonians $H_S^{(1)}$ and $H_S^{(2)}$.

We start by using a standard data-processing-inequality induced by the partial trace, namely

$$D^2(\rho_S(t, H_S^{(1)}), \rho_S(t, H_S^{(2)}) \leq D^2(\rho_{SM}(t, H_{tot}^{(1)}), \rho_{SM}(t, H_{tot}^{(2)}))\ . \tag{B.3}$$

Secondly, we consider the local metric properties of D. Namely, as $D(\rho, \sigma)$ corresponds to the geodesics length of the metrics (A.13) integrated on the set of normalized density matrices, it follows that any other trajectory with the same initial and final points will be longer,

$$D(\rho, \sigma)^2 \leq \frac{1}{4}\int_0^1 \mathrm{d}s\langle\langle\dot{\eta}(s)|\dot{\eta}(s)\rangle\rangle|_{\eta(s)}\quad \text{for any trajectory satisfying}\quad \eta(s=0) = \rho,\ \eta(s=1) = \sigma. \tag{B.4}$$

For our purposes we can specify this relation to trajectories of states corresponding to different evolutions induced by H_S time τ, that is $\eta(s) = \rho_S(\tau, H_s)$ with $H_{s=0} = H_S^{(1)}$ and $H_{s=1} = H_S^{(2)}$, from which

$$D^2(\rho_{SM}(\tau, H_S^{(1)}), \rho_{SM}(\tau, H_S^{(2)})) \leq \frac{1}{4}\int \mathrm{d}s\langle\langle\partial_s\rho_{SM}(\tau, H_s)|\partial_s\rho_{SM}(\tau, H_s)\rangle\rangle_{\rho_{SM}(\tau, H_s)}\ . \tag{B.5}$$

Taking specifically $H_s = H_1 + s(H_2 - H_1)$ we can use the known bound [31, 34]

$$\langle\langle\partial_s\rho_{SM}(\tau, H_s)|\partial_s\rho_{SM}(\tau, H_s)\rangle\rangle_{\rho_{SM}(\tau, H_s)} \leq \frac{\tau^2\|H_S^{(1)} - H_S^{(2)}\|^2}{\hbar^2} \tag{B.6}$$

to finally obtain the bound on the distance between the evolved states on the total $S + M$ space

$$D^2(\rho_{SM}(\tau, H_S^{(1)}), \rho_{SM}(\tau, H_S^{(2)})) \leq \frac{\tau^2\|H_S^{(1)} - H_S^{(2)}\|^2}{4\hbar^2}\ . \tag{B.7}$$

which bounds as well the distance on S alone

$$D(\rho_S(\tau, H^{(1)}), \rho_S(\tau, H^{(2)})) \leq \frac{\tau\|H_S^{(1)} - H_S^{(2)}\|}{2\hbar} \tag{B.8}$$

via (B.3).

i. Average-in-time, dynamical bound, entanglement

For completeness and clarity we re-derive the known bound (B.6) here for any unitarily-parametrized trajectory of states. More precisely, consider the unitary system-machine dynamics (B.2), which is of the form $\rho_\theta(t) = U_\theta(t)\rho(0)U_\theta^\dagger(t)$ where we let the local Hamiltonian $H_S(\theta)$ to be parametrized by some θ. One can compute the Quantum Fisher information as

$$\mathcal{F}(\rho_\theta) = \langle\langle\partial_\theta\rho_\theta(t)|\partial_\theta\rho_\theta(t)\rangle\rangle|_{\rho_\theta(t)} \tag{B.9}$$

using

$$\partial_\theta\rho_\theta(t) = \partial_\theta U_\theta(t)\rho_\theta(t)U_\theta^\dagger(t) + U_\theta(t)\rho_\theta(t)\partial_\theta U_\theta^\dagger(t) = -i\frac{t}{\hbar}[\tilde{H}_\theta(t), \rho_\theta(t)]\ , \tag{B.10}$$

$$\tilde{H}_\theta(t) := i\frac{\hbar}{t}\partial_\theta U_\theta(t)U_\theta^\dagger(t)\ . \tag{B.11}$$

The explicit expression for $\tilde{H}_\theta(t)$ can thus be computed from (B.2) as

$$\tilde{H}_\theta(t) = \int_0^1 \mathrm{d}s\, U_\theta(t,st)\dot{H}_\theta U_\theta(st,0)U_\theta^\dagger(t,0) = \int_0^1 \mathrm{d}s\, U_\theta(t,st)\dot{H}_\theta U_\theta^\dagger(t,st) := \int_0^1 \mathrm{d}s \dot{H}_\theta^{(s)} \,, \tag{B.12}$$

where we indicate by $\dot{H}_\theta \equiv \partial_\theta H_\theta$ which is constant in time, and we introduced the (t,t') propagator for $t \geq t'$

$$U(t,t') = \mathcal{T} \exp\left\{ \frac{i}{\hbar} \int_{t'}^t \mathrm{d}s \; H_\theta(s) \right\} . \tag{B.13}$$

As $\tilde{H}_\theta(t)$ corresponds to an integral average (B.12), we can use the convexity of the QFI to bound

$$\langle\!\langle \partial_\theta \rho_\theta(t) | \partial_\theta \rho_\theta(t) \rangle\!\rangle|_{\rho_\theta(t)} = \frac{t^2}{\hbar^2} \langle\!\langle [\tilde{H}_\theta(t), \rho_\theta(t)] | [\tilde{H}_\theta(t), \rho_\theta(t)] \rangle\!\rangle|_{\rho_\theta(t)} \leq \int_0^1 \mathrm{d}s \frac{t^2}{\hbar^2} \langle\!\langle [\tilde{H}_\theta^{(s)}, \rho_\theta(t)] | [\tilde{H}_\theta^{(s)}, \rho_\theta(t)] \rangle\!\rangle|_{\rho_\theta(t)} \,. \tag{B.14}$$

The last expression can be explicitly rewritten as

$$\begin{aligned} &\frac{t^2}{\hbar^2} \int_0^1 \mathrm{d}s \; \mathrm{Tr} \left[[U_\theta(t,st)\dot{H}_\theta U_\theta^\dagger(t,st), \rho_\theta(t)] \mathbb{J}_{\rho_\theta}^{-1}(t) [\rho_\theta(t), U_\theta(t,st)\dot{H}_\theta U_\theta^\dagger(t,st)] \right] \\ =&\frac{t^2}{\hbar^2} \int_0^1 \mathrm{d}s \; \mathrm{Tr} \left[[\dot{H}_\theta, \rho_\theta(t-st)] \mathbb{J}_{\rho_\theta(t-st)}^{-1} [\rho_\theta(t-st), \dot{H}_\theta] \right] \\ =&\frac{t^2}{\hbar^2} \int_0^1 \mathrm{d}s \; \mathrm{Tr} \left[[\dot{H}_\theta, \rho_\theta(st)] \mathbb{J}_{\rho_\theta(st)}^{-1} [\rho_\theta(st), \dot{H}_\theta] \right] \,. \end{aligned} \tag{B.15}$$

We thus found a meaningful lemma: *the QFI of a parametric time-dependent unitary evolution generated by $H_\theta(t)$ (with fixed derivative $\partial_\theta H_\theta(t) \equiv \partial_\theta H_\theta =: \dot{H}_\theta$, is bounded from above by the average QFI of unitary rotations generated by $\dot{H}_\theta$ on $\rho(st)$ averaged on all times st smaller than t.*

State-agnostic bound. In the absence of further information the upperbound on the QFI

$$\mathcal{F}(\rho_\theta) \leq \frac{t^2}{\hbar^2} \int_0^1 \mathrm{d}s \; \mathrm{Tr} \left[[\dot{H}_\theta, \rho_\theta(st)] \mathbb{J}_{\rho_\theta(st)}^{-1} [\rho_\theta(st), \dot{H}_\theta] \right] \tag{B.16}$$

can finally be bounded using again the convexity of the QFI and noticing that each term is bounded the pure state QFI, that is the variance of $\dot{H}_\theta$ [31]

$$\mathrm{Tr} \left[[\dot{H}_\theta, \rho_\theta(st)] \mathbb{J}_{\rho_\theta(st)}^{-1} [\rho_\theta(st), \dot{H}_\theta] \right] \leq \sum_k p_k \langle \psi_k | \Delta^2 \dot{H}_\theta^2 | \psi_k \rangle \tag{B.17}$$

where $\rho_\theta(st) = \sum_k |\psi_k\rangle\langle\psi_k|$. Finally, the variance of any operator O is bounded by $\|O\|^2/4$, leading to Eq. (B.7).

A comment on entanglement Consider now the case of n-partite Hamiltonian acting on S, that is of the form

$$\partial_\theta H_\theta = \sum_{i=1}^n h_i \tag{B.18}$$

in which each $h_i \equiv \mathbb{I}_1 \otimes \mathbb{I}_2 \otimes \cdots \otimes h_i \otimes \mathbb{I}_{i+1} \otimes \cdots \otimes \mathbb{I}_n \otimes \mathbb{I}_M$ acts locally on the i-th site of the system S, and trivially on M. Suppose now that ρ_θ in (B.16) is k-separable, meaning that it can be written as the convex sum of states with at most k-partite entanglement on S,

$$\rho_\theta = \sum_\alpha \rho_{SM_{\alpha_1}}^{(\alpha_1)} \otimes \rho_{SM_{\alpha_2}}^{(\alpha_2)} \otimes \ldots \,. \tag{B.19}$$

Here, α is a label and SM_{α_i} is the i-th set of a partition of the total $S+M$ system, containing at most k sites of S. It follows then that the QFI $\mathcal{F}_\theta(\rho_\theta)$ is bounded, via convexity, to

$$\frac{\hbar^2}{t^2} \mathcal{F}_\theta(\rho_\theta) \leq \sum_{\vec{\alpha}} p_{\alpha_1^{(i_1)}, \alpha_2^{(i_2)}, \ldots} \left\langle \psi_{\alpha_1^{(i_1)}} \right| \left\langle \psi_{\alpha_2^{(i_2)}} \right| \ldots \Delta^2 \left(\sum_i h_i \right) \left| \psi_{\alpha_1^{(i_1)}} \right\rangle \left| \psi_{\alpha_2^{(i_2)}} \right\rangle \ldots \tag{B.20}$$

where the $\left|\psi^{(i_j)}_{\alpha_j}\right\rangle$ are the eigenvectors of the states ρ_{α_j}, and $p_{\alpha_1^{(i_1)},\alpha_2^{(i_2)},\dots}$ the corresponding probabilities. From convexity and the summation property of the variance for factorized states it then follows the bound

$$\frac{\hbar^2}{t^2}\mathcal{F}_\theta(\rho_\theta) \leq \sum_{\vec{\alpha}} p_{\alpha_1,\alpha_2,\dots}\frac{1}{4}\left(\|\sum_{i\in S_{\alpha_1}} h_i\|^2 + \|\sum_{i\in S_{\alpha_2}} h_i\|^2 + \dots\right) . \tag{B.21}$$

Finally, we notice that this last quantity scales at most as $k^2 \cdot \lceil \frac{n}{k} \rceil$, where k is the maximum size (in sites) of the sets S_{α_i}. In particular, if all the h_i have the same spectral seminorm $\|h_i\| \equiv \|h\| \; \forall i$ (as, for example, in the case of a uniform field), we find

$$\mathcal{F}_\theta(\rho_\theta) \leq \frac{t^2}{\hbar^2} k^2 \left\lceil \frac{n}{k} \right\rceil \frac{\|h\|^2}{4} \tag{B.22}$$

which is valid for k-separable ρ_θ.

C. GENERAL FRAMEWORK AND PLANCKIAN BOUND DERIVATION

In this appendix we provide a complete formal description of the thermalization framework used in our work, and the following derivation of the general Planckian bound (6) on thermalization time.

In C i we formalize the notion of (quantum) thermalization that we use in the main text – specifically, requirement 2 – and include the possibility of a finite time-resolution in the observation of the thermal state.

In C ii we provide the derivation of our main bound (6).

i. A weak nontrivial notion of thermalization

We expect thermalization to be a dynamical mechanism bringing a system close to its thermal ensemble, when in contact with a much larger environment. That is, at some time τ of the dynamics, one should have

$$\rho_S(\tau, H_S) \approx \frac{e^{-\beta H_S}}{\mathrm{Tr}\left[e^{-\beta H_S}\right]} =: \omega(\beta, H_S) \tag{C.1}$$

in some mathematically well-defined sense, for the proposed temperature $T = (k_B\beta)^{-1}$.

First of all, we require the dynamics to be described by standard nonrelativistic quantum mechanics (Requirement 1), that is for some dilation of the system+machine the dynamics is closed and unitary, possibly time-dependent, i.e. governed by the Schrödinger equation

$$\frac{d}{dt}\rho_{SM} = -\frac{i}{\hbar}[H_S + V_{SM}(t) + H_M(t), \rho_{SM}(t)] \tag{C.2}$$

from which the dynamics can be integrated as (B.2)

$$\rho_{SM}(t) = U(t)\rho_{SM}(0)U^\dagger(t) \; , \tag{C.3}$$

$$\rho_S(t) = \mathrm{Tr}_M \rho_{SM}(t) \; . \tag{C.4}$$

Two remarks here:

1) Notice that the degrees of freedom of the environment/machine M might include also d.o.f. of the same particle, for example, the position x of a spin-$\frac{1}{2}$ particle, that only thermalizes in the 2-dimensional space of the spin d.o.f..

2) Notice as well that in standard setups one could have an uncorrelated initial state of the form $\rho_{SM}(0) = \rho_S(0) \otimes \rho_M(0)$, however such assumption is not necessary to our purposes.

We now turn to formalizing the notion of thermalization. We make the weakest possible assumptions on the specifics of Eq. (C.1):

1. Nontrivial local thermalization: in order to have a proper thermalization process[2], (C.1) should be valid at least for more than one single Hamiltonian. In order to derive a nontrivial Planckian bound, it will be enough to

[2] As discussed in the main text, thermalization for a single value of the local H_S can be trivially obtained by storing copies of $\omega(\beta, H_S)$ in advance.

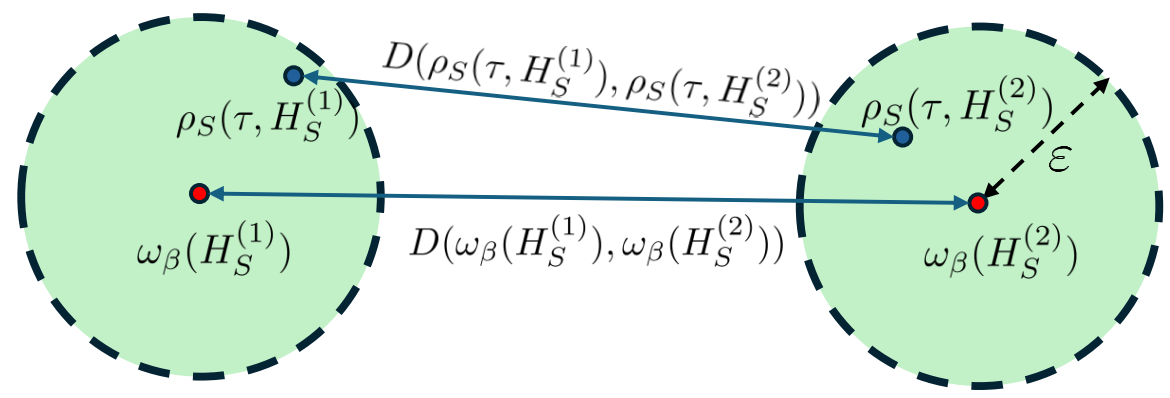

FIG. S1. Illustration of the triangular inequality (C.9). As the dynamics $\rho_S(\tau, H_S)$ is required to follow the local thermal states up to error ε, the distance between $\rho_S(\tau, H_S^{(1)})$ and $\rho_S(\tau, H_S^{(2)})$ is bounded from below.

consider H_S taking two values, $H_S^{(1)}$ and $H_S^{(2)}$ (cf. C ii). However, for simplicity we will often consider in some continuous set of Hamiltonian $\mathcal{H}$, such as the neighborhood $\mathcal{H} \equiv \mathcal{B}_\delta$ of a given $\bar{H}_S$

$$\mathcal{B}_\delta : \{H_S \big| \, \|H_S - \bar{H}_S\| \leq \delta\} \tag{C.5}$$

according to some norm.

2. Thermalization is approximate: for all values of $H_S \in \mathcal{H}$, one should have

$$D(\rho_S(\tau, H_S), \omega(\beta, H_S)) \leq \varepsilon \quad \forall H_S \in \mathcal{H} \ , \tag{C.6}$$

according to some notion of distance D. Here ε thus indicates the admitted error of the thermalization process.

3. Time-resolution: finally we also allow for a non-exact time-resolution of the measurement devices. That is, we assume that rather than $\rho_S(\tau, H)$, the state sampled by the machine's user is given by a time-average

$$\tilde{\rho}_S(\tau, H_S) := \int_0^\tau \mathrm{d}t \; \mu(t) \rho_S(t, H_S) \quad \text{for some measure } \int_0^\tau \mathrm{d}t \; \mu(t) = 1 \ . \tag{C.7}$$

These conditions correspond to (a more general version of) machine requirement 2 in the main text.

Definition of thermalization. Putting together the conditions above, we arrive to the formal statement of what is thermalization to the canonical ensemble, in its weakest form, that is we require

$$D(\tilde{\rho}_S(\tau, H_S), \omega_\beta(H_S)) \leq \varepsilon \quad \forall H_S \in \mathcal{H} \ . \tag{C.8}$$

ii. Formal bound

The Planckian bound on thermalization is the consequence of a triangular inequality associated to (C.8), namely for any two values $H_S^{(1)}$ and $H_S^{(2)}$ in the set of thermalizing Hamiltonians $\mathcal{H}$, it follows from (C.8) that (see Fig. S1)

$$D(\tilde{\rho}_S(\tau, H_S^{(1)}), \tilde{\rho}_S(\tau, H_S^{(2)})) \geq D(\omega(\beta, H_S^{(1)}), \omega(\beta, H_S^{(2)})) - 2\varepsilon \ . \tag{C.9}$$

This is the starting point of our derivation. We now pass to upper-bounding the left-hand side of (C.9). If the chosen distance D features a joint-convexity property, we can use it to take into account the time-resolution of the measurement devices (C.7), that is, from joint convexity of D^2 it follows

$$D^2(\tilde{\rho}_S(t, H_1), \tilde{\rho}_S(t, H_2)) \leq \max_{0 \leq t \leq \tau} D^2(\rho_S(t, H_S^{(1)}), \rho_S(t, H_S^{(2)})) \tag{C.10}$$

Finally, we use a bound of the form (as is the case (B.8) with our choice of D and $\| \ldots \|$)

$$D^2(\rho_S(t, H_S^{(1)}), \rho_S(t, H_S^{(2)})) \leq \frac{t^2 \|H_1 - H_2\|^2}{4\hbar^2} \ . \tag{C.11}$$

for the norm $\|\ldots\|$. It then follows by concatenating (C.9)-(C.10)-(C.11), and noticing $t \leq \tau$, that

$$\tau \geq \hbar \frac{2D(\omega_\beta(H_S^{(1)}), \omega_\beta(H_S^{(2)})) - 4\varepsilon}{\|H_S^{(1)} - H_S^{(2)}\|} = \beta\hbar \frac{2D(\omega_\beta(H_S^{(1)}), \omega_\beta(H_S^{(2)})) - 4\varepsilon}{\beta\|H_S^{(1)} - H_S^{(2)}\|} \ . \tag{C.12}$$

This bound needs to be satisfied in the whole range of Hamiltonians for which we request the thermalization to be valid, i.e. $\forall H_S^{(1)}, H_S^{(2)} \in \mathcal{H}$. We therefore have in general the relation

$$\tau \geq \beta\hbar\, \chi(\mathcal{H}, \varepsilon) \ , \tag{C.13}$$

where

$$\chi(\mathcal{H}, \varepsilon) = \max_{H_S^{(1)}, H_S^{(2)} \in \mathcal{H}} \frac{2D(\omega_\beta(H_S^{(1)}), \omega_\beta(H_S^{(2)})) - 4\varepsilon}{\beta\|H_S^{(1)} - H_S^{(2)}\|} \ . \tag{C.14}$$

This is our main result, presented also in the main text (6). A few remarks are in order: first, notice that χ solely depends on βH_S rather than H_S itself. This makes it dimensionless and, in general, (as we prove below) finite. More specifically, χ can be seen to depend on the set Ω spanned by the thermal states obtained from Hamiltonians in $\mathcal{H}$ (at temperature β^{-1}). Moreover, notice that in general for the bound to be nontrivial, 2ε should be smaller than $D(\omega(\beta, H_S^{(1)}), \omega(\beta, H_S^{(2)}))$ for some choice of thermal states in the region $\mathcal{H}$ under scrutiny.

In this work, we choose $D \equiv D_A$ to be the Bures angle (A.2), and $\|\ldots\|$ the spectral seminorm (A.14), for which it holds the upperbound (B.8). Notice that with this choice of normalization, the maximum distance between two states is given by $\pi/2$, therefore the tolerated ε should be between 0 and

$$\varepsilon_{\max} = \frac{\pi}{4} \tag{C.15}$$

in order to yield a nontrivial bound. In fact, for any two given (thermal) states $\omega(\beta, H_S^{(1)})$ and $\omega(\beta, H_S^{(2)})$, it is possible to find a state $\bar{\omega}$ that satisfies $D(\omega(\beta, H_S^{(1)}), \bar{\omega}) \leq \pi/4$ and $D(\omega(\beta, H_S^{(2)}), \bar{\omega}) \leq \pi/4$. The instantaneous preparation of $\bar{\omega}$ would then yield a $\varepsilon_{\max}$-thermalization machine for $\mathcal{H} \equiv \{H_S^{(1)}, H_S^{(2)}\}$ (as well as for all the Hamiltonians generating thermal states $\varepsilon_{\max}$-close to $\bar{\omega}$). In the qubit case, the maximally mixed state $\mathbb{I}/2$ satisfies $D(\mathbb{I}/2, \rho) \leq \varepsilon_{\max}\ \forall \rho$.

D. LOWERBOUNDS TO $\tilde{\chi}(\rho)$ (LOCALLY-EXACT THERMALIZATION)

The QFI of states in exponential forms

$$\rho_\theta = \frac{e^{-X_\theta}}{\mathrm{Tr}\left[e^{-X_\theta}\right]} \tag{D.1}$$

(such as Gibbs states in natural units where $X = \beta H$), is given by substituting the exponential derivative

$$\partial_\theta \rho_\theta = \left(\int_0^1 \mathrm{d}s \frac{e^{-sX_\theta} \partial_\theta X_\theta e^{-(1-s)X_\theta}}{\mathrm{Tr}\left[e^{-X_\theta}\right]} \right) - \frac{e^{-X_\theta}}{\mathrm{Tr}\left[e^{-X_\theta}\right]} \mathrm{Tr}\left[\partial_\theta X_\theta \frac{e^{-X_\theta}}{\mathrm{Tr}\left[e^{-X_\theta}\right]} \right] \tag{D.2}$$

inside (A.13). The resulting expression can be written, in the basis of eigenstates of $\rho_\theta = \sum_i p_{\theta_i} |\psi_{\theta_i}\rangle\langle\psi_{\theta_i}|$ as (cf. [32])

$$\mathcal{F}_\theta = \sum_{ij} \frac{2(p_i - p_j)^2}{(\ln p_i - \ln p_j)^2 (p_i + p_j)} X'_{ij} - \left(\sum_i p_i X'_{ii} \right)^2 \quad \text{where} \quad X'_{ij} := \langle \psi_{\theta_i} | \, \partial_\theta X_\theta \, | \psi_{\theta_j} \rangle \ . \tag{D.3}$$

In the limit of locally-exact thermalization described in , the adimensional factor $\tilde{\chi}$ in the Planckian bound $\tau \geq \tau_{\mathrm{Pl}} \cdot \tilde{\chi}$ simply becomes the maximisation of $\sqrt{\mathcal{F}_\theta}$ over all possible directions of perturbation X' with spectral norm $\|X'\| = 1$. We thus want to estimate

$$\tilde{\chi}(\rho) := \max_{\|X'\|=1} \sqrt{ \sum_i p_i {X'}_{ii}^2 - (\sum_i p_i {X'}_{ii})^2 + \sum_{ij} |X'_{ij}|^2 \frac{2(p_i - p_j)^2}{(\ln p_i - \ln p_j)^2 (p_i + p_j)} } \ .$$

In the following, we provide several lower bounds to $\tilde{\chi}$ based on different possible choices of X'.

(i) Diagonal bound: When considering only diagonal perturbations ($X'_{ij} = 0$ (for $i \neq j$)) it is relatively straightforward to maximise the corresponding classical variance and obtain

$$\tilde{\chi}(\rho) \geq \tilde{\chi}(\rho)^{\text{clas}} = \sqrt{p^*(1-p^*)} \tag{D.4}$$

which is obtained choosing e.g. $X_{ii} = 1$ for a subset $i \in I$ of the microstates in the diagonal decomposition of ρ, having total probability $P(I) = p^*$, ($X_{ii} = 0$ for $i \notin \mathcal{I}$ to guarantee $\|X\|$=1). That is, for any to coarse graining of the thermal state's populations in $\{p^*, 1-p^*\}$ one has a corresponding inequality (D.4). Clearly the value of the coarse-grained probability that is closest to 1/2 will yield the best bound.

For example, notice that whenever the ground state probability p_0 is below $p_0 \leq \frac{2}{3}$, one can always find a coarse graining such that $\frac{1}{3} \leq p^* \leq \frac{2}{3}$, and therefore $\chi \geq \frac{\sqrt{2}}{3} \sim 0.47$.

(ii) Coherent bound: One can consider a different subset of (non diagonal) perturbations of the form $X'_{ij} = \frac{1}{2}$ (again guaranteeing $\|X'\| = 1$) for any set of non-overlapping pairs of indices, obtaining

$$\tilde{\chi}^2(\rho) \geq \sum_{\text{couples}\{ij\}} \frac{1}{4} \frac{4(p_i - p_j)^2}{(\ln p_i - \ln p_j)^2 (p_i + p_j)} \tag{D.5}$$

Notice that for fixed "fraction of population" used, one should privilege to couple probabilities that are close to each other. In the limit of very-close populations each term tends to $\frac{1}{2} p_i$. Therefore the maximum is again for $p_i = 1/2$ and is the same as the classical maximum above. However, for large gaps/ very pure states it is useful to see that

$$\sum_{\text{couples}\{ij\}} \frac{1}{4} \frac{4(p_i - p_j)^2}{(\ln p_i - \ln p_j)^2 (p_i + p_j)} \geq \frac{(p_0 - p_1)^2}{(\ln p_0 - \ln p_1)^2} \geq \frac{(2p_0 - 1)^2}{(\beta\Delta)^2} \geq \frac{(2p_0 - 1)^2}{(\ln p_0 - \ln(1-p_0) + \ln(d-1))^2} \,. \tag{D.6}$$

(iii) Full Qubit bound: In the case of a single Qubit one solve the maximisation exactly and interpolate between the previous two bounds by choosing up to symmetry in the Bloch-sphere $X' = \frac{1}{2}(\cos(\theta)\sigma_z + \sin(\theta)\sigma_x)$ and obtain

$$\tilde{\chi}^2(\rho)^{\text{Qbit}} = \max_{\theta} \left(\cos(\theta)^2 p(1-p) + \sin(\theta)^2 \frac{(2p-1)^2}{(\ln p - \ln(1-p))^2} \right) \,. \tag{D.7}$$

We see clearly that the second addend is always bigger than the previous, therefore $\sin(\theta)^2 = 1$ is preferable, yielding the dashed green line bound in Fig. S2.

E. LOWER BOUNDS TO $\chi(\mathcal{H}, \varepsilon)$ (FINITE-SIZE/FINITE-ERROR THERMALIZATION)

In this section we provide bounds to $\chi(\mathcal{H}, \varepsilon)$ (C.14) for finite sizes of error and Hamiltonian set $\mathcal{H}$ for which the approximate thermalization holds. In the main text and in the following we take for simplicity $\mathcal{H}$ to be a ball $\mathcal{B}_\delta$ of Hamiltonians centered in some $\bar{H}_S$ and with spectral norm radius δ, thus $\chi(\mathcal{H}, \varepsilon) \equiv \chi(\bar{H}_S, \delta, \varepsilon)$. Physically speaking, as opposed to the limit $\varepsilon \ll \delta \to 0$ (infinitely precise and infinitely local thermalization), here we are crucially considering the regime in which the error ε is strictly finite, and as such thermalization is also required to be valid in a finite region of Hamiltonians/thermal states. As noticed above, the adimensional quantity χ only depends on the thermal state $\omega(\beta, H_S)$ statistics and the adimensional exponent βH_S. For this reason we will simplify the notation in the following and consider temperature units in which $\beta = 1$.

We consider Hamiltonians contained in a ball $\mathcal{B}_\delta : \{H_S | \|H_S - \bar{H}_S\| \leq \delta\}$ (clearly, for any two Hamiltonians in this set we have $\|H_S^{(1)} - H_S^{(2)}\| \leq 2\delta$). To get a lowerbound to $\chi(\bar{H}_S, \delta, \varepsilon)$, we take the subset ansatz of the form

$$H_S^{(1)} = \bar{H}_S + \alpha\kappa \,, \quad H_S^{(2)} = \bar{H}_S - (1-\alpha)\kappa \,, \tag{E.1}$$

where

$$0 \leq \alpha \leq 1 \,, \quad \|\kappa\| = \delta \,, \tag{E.2}$$

and more in particular we consider κ to be diagonal in the basis of $\bar{H}_S$, with eigenvalues k_i and of the form

$$\begin{aligned} \kappa_i &= 0 \;\forall i \in I_0 \,, \\ \kappa_i &= \delta \;\forall i \in I_1 \,. \end{aligned} \tag{E.3}$$

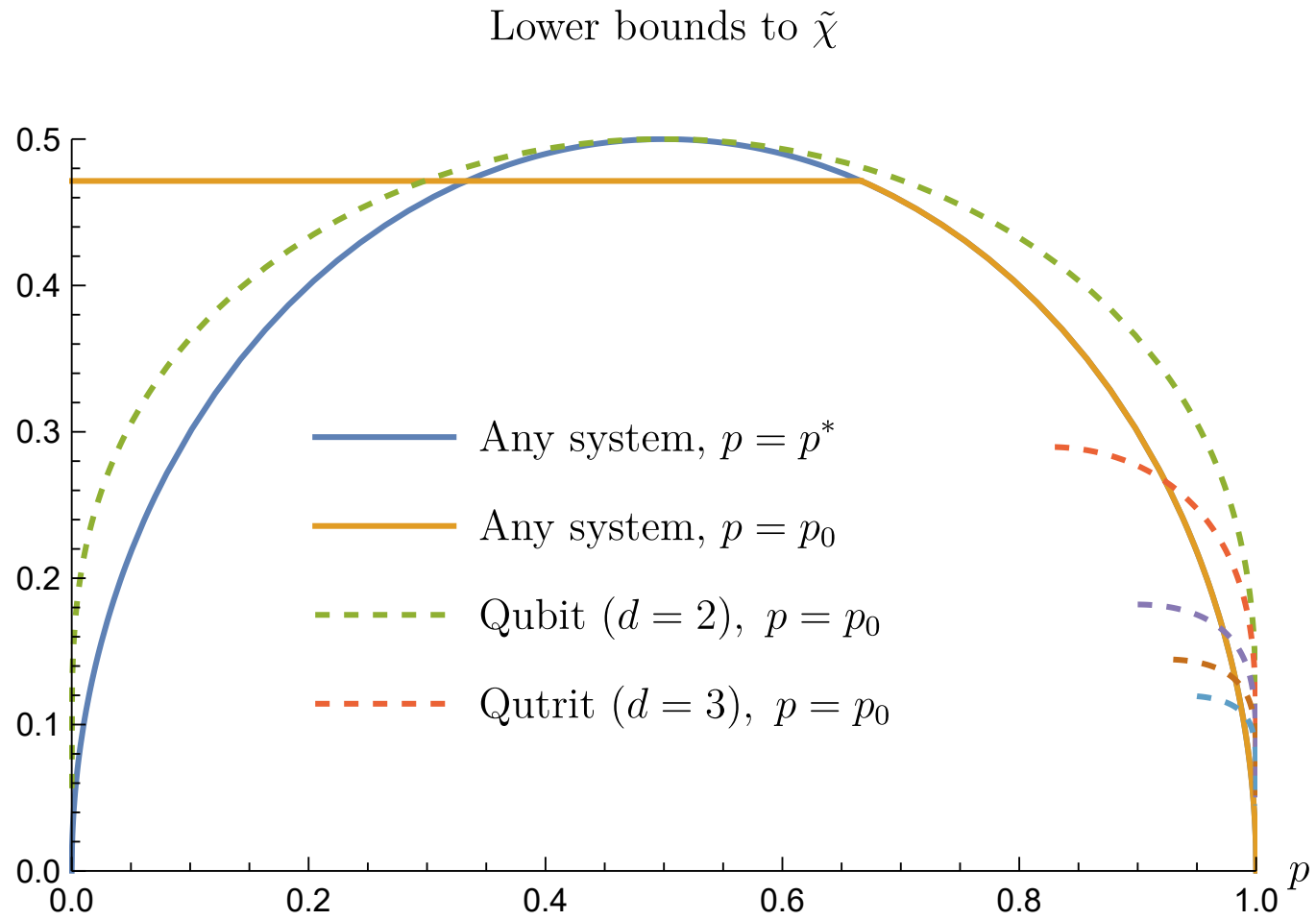

FIG. S2. **Bounds for locally-exact thermalization.** In the limit locally-exact thermalization (see text), our main bound (6) reduces to $\tau \geq \tau_{\rm P1} \cdot \tilde{\chi}$ (7), which only depends on the local thermal state considered. In the plot, different lower bounds to $\tilde{\chi}$ are shown. We find that $\tilde{\chi} \geq \sqrt{p^*(1-p^*)}$ (blue line) for all possible coarse-grainings of the thermal states' populations in two sets with total probabilities $\{p^*, 1-p^*\}$. The maximum of such function is obtained for $p^* = 1/2$ which is easily approximated in noncritical large systems. Moreover this immediately yields $\tilde{\chi} \geq \sqrt{2}/3 \sim 0.47$ for any thermal state with ground state probability p_0 smaller than $p_0 \leq 2/3$ (yellow line). In terms of spectral gap Δ, notice that ground state probability p_0 always satisfies $p_0 \leq \frac{1}{1+e^{-\beta\Delta}}$, thus $\tilde{\chi} \geq \sqrt{e}/(1+e) \sim 0.44$ for $\beta\Delta \leq 1$. For very large values of p_0, it is possible to show that $\tilde{\chi} \geq (2p_0 - 1)/(\beta\Delta) \geq (2p_0-1)/(\ln((d-1)p_0/(1-p_0)))$, which yields the dashed lines in the plot (for increasing dimension, in the plot $d = 2, 3, 10, 30, 100$). A full analytical optimization of $\tilde{\chi}$ is possible for qubits (green dashed line): given $X' = \frac{1}{2}(\cos(\theta)\sigma_z + \sin(\theta)\sigma_x)$, the transversal bound $\sin\theta = 1$ is optimal and always larger than the diagonal one, cf. (D.6).

That is κ has only two possible eigenvalues, it represents a shift of a subset (I_1) of the eigen-energies E_i of $\bar{H}_S$. Moreover the choice (E.1) guarantees that both $H_S^{(1)}$ and $H_S^{(2)}$ are in $\mathcal{B}_\delta$ and $\kappa \equiv H_S^{(1)} - H_S^{(2)}$.

With such choice it is possible to compute the value of the fidelity (A.1) entering the definition of χ (C.14) via the Bures angle $D(\rho,\sigma) = \arccos\sqrt{F(\sigma,\rho)}$, that is

$$\sqrt{F(\omega^{(1)},\omega^{(2)})} = \sum_i \frac{e^{-(E_i+\alpha\kappa_i)/2}}{\sqrt{\sum_j e^{-(E_j-(1-\alpha)\kappa_j)}}} \frac{e^{-(E_i+\alpha\kappa_i)/2}}{\sqrt{\sum_j e^{-(E_j-(1-\alpha)\kappa_j)}}} \tag{E.4}$$

$$= \frac{q_0 + q_1 e^{(1-2\alpha)\delta/2}}{\sqrt{q_0 + q_1 e^{-\alpha\delta}}\sqrt{q_0 + q_1 e^{(1-\alpha)\delta}}} \tag{E.5}$$

where to compute the second line we defined

$$q_0 = \sum_{i\in I_0} e^{-E_i}\ , \quad q_1 = \sum_{i\in I_1} e^{-E_i}\ . \tag{E.6}$$

This allows to re-express the fidelity in terms of the coarse-grained probabilities of the thermal state of $\bar{H}_S$, that is given $p^* = q_0/(q_0+q_1)$ and $1-p^* = q_1/(q_0+q_1)$, namely

$$\sqrt{F(\omega^{(1)},\omega^{(2)})} = \frac{p^* + (1-p^*)e^{(1-2\alpha)\delta/2}}{\sqrt{p^* + (1-p^*)e^{-\alpha\delta}}\sqrt{p^* + (1-p^*)e^{(1-\alpha)\delta}}}\ , \tag{E.7}$$

We thus find a continuous familty of bounds for $\mathcal{H} = \mathcal{B}_\delta$ as a function of all possible coarse-grainings p^* of the populations of $\omega(\beta,\bar{H}_S)$

$$\chi(\mathcal{B}_\delta(\omega(\beta,\bar{H}_S)),\varepsilon) \geq \frac{1}{\delta}\left(2\arccos\left(\frac{p^* + (1-p^*)e^{(1-2\alpha)\delta/2}}{\sqrt{p^* + (1-p^*)e^{-\alpha\delta}}\sqrt{p^* + (1-p^*)e^{(1-\alpha)\delta}}}\right) - 4\varepsilon\right)\ . \tag{E.8}$$

In Figure S3 we plot the bounds attainable by choosing specific (α,δ)-configurations described in the following.

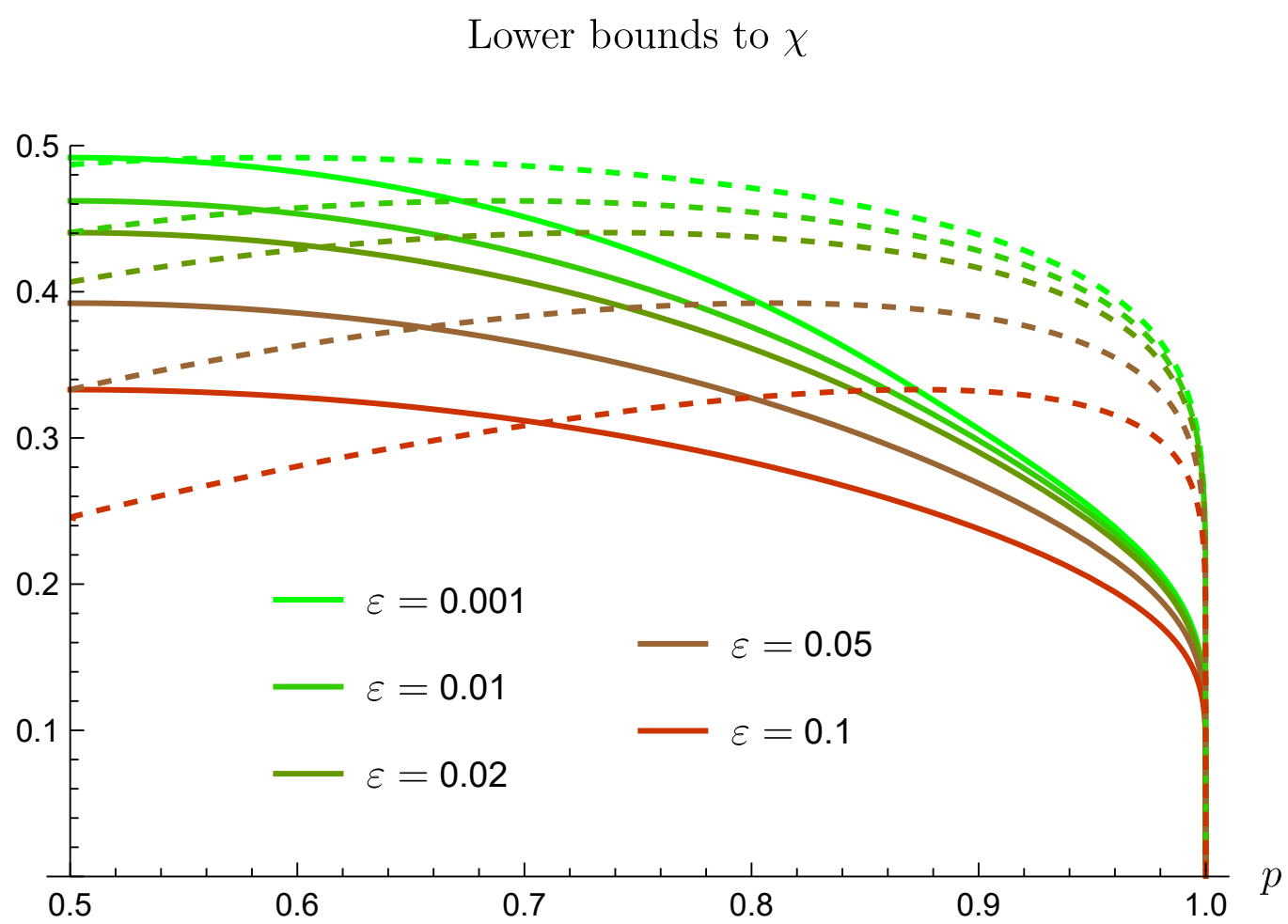

FIG. S3. Lower bounds to $\chi(\bar{H}_S, \varepsilon)$ obtained by considering set of ansatz Hamiltonians (E.1) belonging to $\mathcal{B}_\delta$. Lower bounds to χ are given as a function of the coearse-grained probability $p = p^*$ in the thermal state defined by $\bar{H}_S$, by maximising over δ the expression (E.8). In particular we consider "radial variations" (dashed lines, $\alpha = 0$ or $\alpha = 1$), and "diametral variations" (continuous lines, $\alpha = 1/2$) around $\bar{H}_S$.

a. Radial variations around $\bar{H}_S$. Radial variations around $\bar{H}_S$ correspond to the choice $\alpha = 1$ (or $\alpha = 0$), that is when either $H_S^{(1)}$ or $H_S^{(2)}$ coincide with the center of the ball $\mathcal{B}_\delta$.

b. Diametral variations around $\bar{H}$. Diametral variations around $\bar{H}_S$ correspond to the choice $\alpha = 1/2$, that is when either $H_S^{(1)} - \bar{H}_S = -(H_S^{(2)} - \bar{H}_S)$. That is such variations are symmetric around the center of the ball $\mathcal{B}_\delta$.

c. Interpolation between the radial and diametral case. Numerical results indicate that diametral perturbations ($\alpha = 1/2$) yield the best lower bounds for systems in which it is possible to find $p^* \approx 1/2$, whereas radial perturbations ($\alpha = 0, 1$) provide better bounds in the (ground state) limit $p^* \to 1$. Intuitively, this reflects the fact that close to mixed states the QFI (and its Bures angle integral) is maximal and isotropic, while for thermal states close to being pure ($\beta\Delta \gg 1$) there is no sensitivity in increasing the energy of the excited levels, as the state is already close to its ground microstate (the susceptibility to such energy variations goes to zero exponentially), whereas increasing the energy of the ground state allows a nontrivial variation of the thermal state.

In the main text Fig. 2, we plot the numerical maximisation of (E.8) for given p^* and ε over α and δ, which interpolates between radial and diametral perturbations in general, providing the highest lower bound in each case.

d. Accuracy-generality tradeoff As discussed above, when introducing the possibility of a finite error ε in the thermalization process, the size of the set $\mathcal{H}$ of thermalizing Hamiltonian needs to be sufficiently large in order to provide In Fig. S4 we plot the lower bound (E.8) obtained for $p^* = 0.5$ and optimized over δ for each value of $0 \leq \varepsilon \leq \varepsilon_{\max}$. We notice how it smoothly interpolates between $\chi \geq 1/2$ (for $\varepsilon = 0$) and $\chi \geq 0$ for $\varepsilon = \pi/2$. For each error we also plot the corresponding δ needed to obtain the lower bound. As expected, as bigger errors are tolerated by the user, more distinguishable thermal states should be asked to the machine.

F. THE OPTIMAL THERMALIZATION MACHINE FOR TWO HAMILTONIANS

We now consider machines that thermalize, i.e. prepare Gibbs states $\rho_S := \omega(\beta, H_S^{(1)})$ and $\sigma_S := \omega(\beta, H_S^{(2)})$ of, two fixed but unknown Hamiltonians $H_S^{(1)}$ or $H_S^{(2)}$.

By Uhlmann's theorem [ii], the two states ρ_S and σ_S admit purifications $|\Psi_\rho\rangle_{SM}$ and $|\Psi_\sigma\rangle_{SM}$ on an extended system SM, i.e. $\mathrm{Tr}_M |\Psi_\rho\rangle\langle\Psi_\rho|_{SM} = \rho_S$ and $\mathrm{Tr}_M |\Psi_\sigma\rangle\langle\Psi_\sigma|_{SM} = \sigma_S$, such that

$$|\langle\Psi_\rho|\Psi_\sigma\rangle|^2 = F(\rho, \sigma) \quad \Longleftrightarrow \quad \arccos|\langle\Psi_\rho|\Psi_\sigma\rangle| = D(\rho, \sigma). \tag{F.1}$$

By definition, a machine preparing the purifications $|\Psi_\rho\rangle$, $|\Psi_\sigma\rangle$ fulfills the thermalization task.

Consider a simple machine (achieving optimal Hamiltonian discrimination [36]) with sets the "interaction" term to

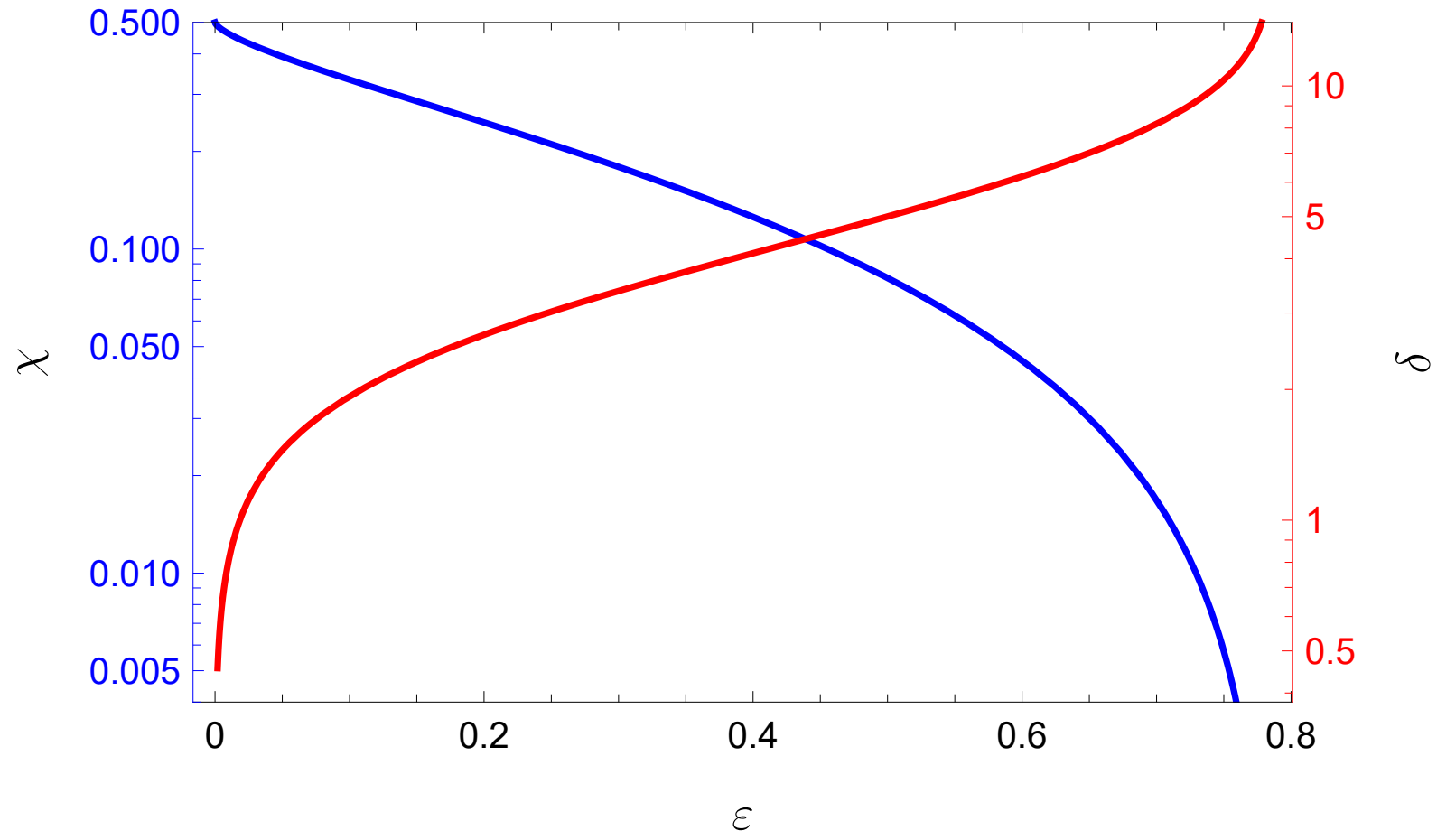

FIG. S4. **Accuracy-generality tradeoff.** For a thermal state in which a 50/50 population split ($p^* = 0.5$) can be identified the $\pm\delta$ energy shift offers lowerbounds to χ that smoothly interpolate between $\chi \geq 0.5$ at zero error (only infinitesimal δ required, namely, locally-exact thermalization) and progressively smaller lower bounds at larger errors ($\chi \geq 0$ only in the limit of 100% thermalization error, namely $\varepsilon = \varepsilon_{\max} = \pi/4$).

$V_{SM} = V_S = -H_S^{(1)}$, and prepares the system in the initial state

$$|\psi(0)\rangle_S = \frac{1}{\sqrt{2}}(|\uparrow\rangle + |\downarrow\rangle)_S \tag{F.2}$$

where $|\uparrow(\downarrow)\rangle$ are the eigenstates of of $\kappa = H_S^{(2)} - H_S^{(1)}$ corresponding to its maximal (minimal) eigenvalues $\lambda_{\uparrow(\downarrow)}$. After time τ it prepares the system in the two possible states

$$|\psi_1(\tau)\rangle = e^{-i\frac{\tau}{\hbar}(H_S^{(1)} - H_S^{(1)})}|\psi(0)\rangle = \frac{1}{\sqrt{2}}(|\uparrow\rangle + |\downarrow\rangle) \tag{F.3}$$

$$|\psi_2(\tau)\rangle = \frac{1}{\sqrt{2}}e^{-i\frac{\tau}{\hbar}(H_S^{(2)} - H_S^{(1)})}(|\uparrow\rangle + |\downarrow\rangle) = \frac{1}{\sqrt{2}}(e^{-i\frac{\tau}{\hbar}\lambda_\uparrow}|\uparrow\rangle + e^{-i\frac{\tau}{\hbar}\lambda_\downarrow}|\downarrow\rangle) \tag{F.4}$$

corresponding to the tow possible value system Hamiltonians $H_S^{(1)}$ and $H_S^{(2)}$. The overlap of the two states is given by

$$|\langle\psi_1(\tau)|\psi_2(\tau)\rangle|^2 = \left|\frac{e^{-i\frac{\tau}{\hbar}\lambda_\uparrow} + e^{-i\frac{\tau}{\hbar}\lambda_\downarrow}}{2}\right|^2 = \cos^2\left(\frac{\|\kappa\|\tau}{2\hbar}\right). \tag{F.5}$$

Finally, note that by an isometry the two state $|\psi_1(\tau)\rangle_S$ and $|\psi_2(\tau)\rangle_S$ can be mapped (up to an irrelevant phase) to another pair of states

$$U|\psi_1(\tau)\rangle_S|0\rangle_M = |\Psi_\rho\rangle_{SA} \tag{F.6}$$
$$U|\psi_2(\tau)\rangle_S|0\rangle_M = |\Psi_\sigma\rangle_{SA} \tag{F.7}$$

if only and only if the pairs of states have the same overlap $|\langle\psi_1|\psi_2\rangle| = |\langle\Psi_\sigma|\Psi_\rho\rangle| = \sqrt{F}$. To see this formally, note that we can always write

$$|\psi_1(\tau)\rangle_S = |1\rangle_S \qquad e^{i\phi}|\psi_2(\tau)\rangle_S = \sqrt{F}|1\rangle_S + \sqrt{1-F}|2\rangle_S \tag{F.8}$$
$$|\Psi_\rho\rangle_{SM} = |\tilde{1}\rangle_{SM} \qquad e^{i\phi'}|\Psi_\sigma\rangle_{SM} = \sqrt{F}|\tilde{1}\rangle_{SM} + \sqrt{1-F}|\tilde{2}\rangle_{SM} \tag{F.9}$$

with orthogonal states $\langle 1|2\rangle = \langle\tilde{1}|\tilde{2}\rangle = 0$. Now, the isometry $V = |\tilde{0}\rangle\langle 0| + |\tilde{1}\rangle\langle 1|$ gives

$$V|\psi_1(\tau)\rangle_S = |\Psi_\rho\rangle_{SM} \tag{F.10}$$
$$V|\psi_2(\tau)\rangle_S = e^{-i\phi}V(\sqrt{F}|1\rangle_S + \sqrt{1-F}|2\rangle_S) = e^{-i\phi}(\sqrt{F}|\tilde{1}\rangle_{SM} + \sqrt{1-F}|\tilde{2}\rangle_{SM}) = e^{i(\phi'-\phi)}|\Psi_\sigma\rangle_{SM} \tag{F.11}$$

Hence our machine is capable of preparing the two Gibbs state for

$$F(\rho,\sigma) = |\langle\Psi_\rho|\Psi_\sigma\rangle|^2 = |\langle\psi_1(\tau)|\psi_2(\tau)\rangle|^2 = \cos^2\left(\frac{\|\kappa\|\tau}{2\hbar}\right), \text{ i.e.} \tag{F.12}$$

$$\tau = \frac{2\hbar}{\|\kappa\|}\arccos|\langle\Psi_\rho|\Psi_\sigma\rangle| = \frac{2\hbar\, D(\sigma,\rho)}{\|H_S^{(2)} - H_S^{(1)}\|} = \frac{2\hbar\, D(\omega(\beta,H_S^{(1)}),\omega(\beta,H_S^{(2)}))}{\|H_S^{(2)} - H_S^{(1)}\|}. \tag{F.13}$$

Of course it can also work for any longer time $\tau' > \tau$ by storing the two target states in a quantum memory after time τ.

G. RESONANT LEVEL MODEL

Let us consider a single electron quantum dot with Hamiltonian

$$H_S = E_S \hat{a}^\dagger \hat{a}\ , \tag{G.1}$$

where $\hat{a}^\dagger$ is its creation operator, it satisfies the anticommutation relation $\{\hat{a}^\dagger,\hat{a}\} = \mathbb{I}$ and $\{\hat{a},\hat{a}\} = 0$. The energy gap of the two-level system is given by E_S.
Here we will model the "thermalizing machine" as a fermionic bath with Hamiltonian

$$H_M = \sum_{k=1}^{n} \Omega_k \hat{b}_k^\dagger \hat{b}_k\ , \tag{G.2}$$

$\hat{b}_k^\dagger$ is the creation operator of a bath mode with frequency Ω_k. These satisfy the relations $\{\hat{b}_j^\dagger,\hat{b}_k\} = \delta_{jk}\mathbb{I}$, $\{\hat{b}_j,\hat{b}_k\} = \{\hat{a},\hat{b}_k\} = \{\hat{a}^\dagger,\hat{b}_k\} = \{\hat{a},\hat{a}\} = 0$. The machine interacts with the system via tunneling effects $\hat{a}^\dagger\hat{b}_k$ and $\hat{b}_k^\dagger\hat{a}$ with some modes of the machine, consistently with the most general quadratic Hamiltonian – such as standard models describing quantum dots interacting with metallic leads. All in all, the $S+M$ Hamiltonian writes as follows

$$H_{tot}(t) = H_S + H_M + g(t)\sum_{k=1}^{n} \lambda_k \hat{a}^\dagger \hat{b}_k + \lambda_k^* \hat{b}_k^\dagger \hat{a}\ . \tag{G.3}$$

Following the same steps as in Ref. [38] (taking the wide-band and thermodynamic limit $\sum_k |\lambda_k|^2\delta(\Omega-\Omega_k) \to 2\pi$), one can solve the exact dynamics of the total system and compute the occupation probability of the quantum dot $p(t) := \mathrm{Tr}\left[\hat{a}^\dagger\hat{a}\rho(t)\right]$, we obtain

$$p(t) = p(0)e^{-\int_0^t dr g(r)^2} + \frac{1}{2\pi}\int_{-\infty}^{\infty} d\Omega\ f_\beta(\Omega)\left|\int_0^t ds\ g(s)e^{-\frac{1}{2}\int_s^t dr g(r)^2} e^{-i(E_S-\Omega)(t-s)}\right|^2\ , \tag{G.4}$$

where $f_\beta(\Omega) = (1+e^{\beta\Omega})^{-1}$ is the Fermi-Dirac distribution. For this system the thermal state is diagonal, and the norm of the off-diagonal element of $\rho(t)$ evolves as $e^{-\int_0^t dr g(r)^2}$. Since it decreases as fast or faster than $p(t)$ we will only consider initial states of the system which are diagonal.

The main characterization we will investigate for this machine is the coupling $g(t)$. At time τ we disconnect the system from the machine and therefore we have $g(t) = 0$ for $t \geq \tau$ and output $\rho(\tau)$. We will test how close we can get to the Planckian time bound for the set $\mathcal{H} = \{H_S^{(1)}, H_S^{(2)}\}$. In this case the Plackian bound Eq. (6) looses the max, we therefore have

$$\tau \geq \tau_{\mathrm{Pl}} \frac{2D(\omega_\beta(H_S^{(1)}),\omega_\beta(H_S^{(2)})) - 4\varepsilon}{\beta\|H_S^{(1)} - H_S^{(2)}\|}\ . \tag{G.5}$$

Before considering specific functions of time for $g(t)$, let us take the simpler case where it is a constant. In this case, we can solve the integrals in Eq. (G.4) and show that $p(t)$ converges towards the thermal probability of the *total* system as fast as $e^{-\frac{g^2}{2}t}$

$$\lim_{t\to\infty} p(t) = \int_{-\infty}^{+\infty} \frac{d\Omega}{\pi} f_\beta(\Omega)\frac{g^2/2}{g^4/4 + (E-\Omega)^2} = \frac{1}{2} - \frac{1}{\pi}\,\mathrm{Im}\,\Psi\left[\frac{1}{2} + \frac{\beta}{2\pi}\left(\frac{g^2}{2} + iE\right)\right]\ , \tag{G.6}$$

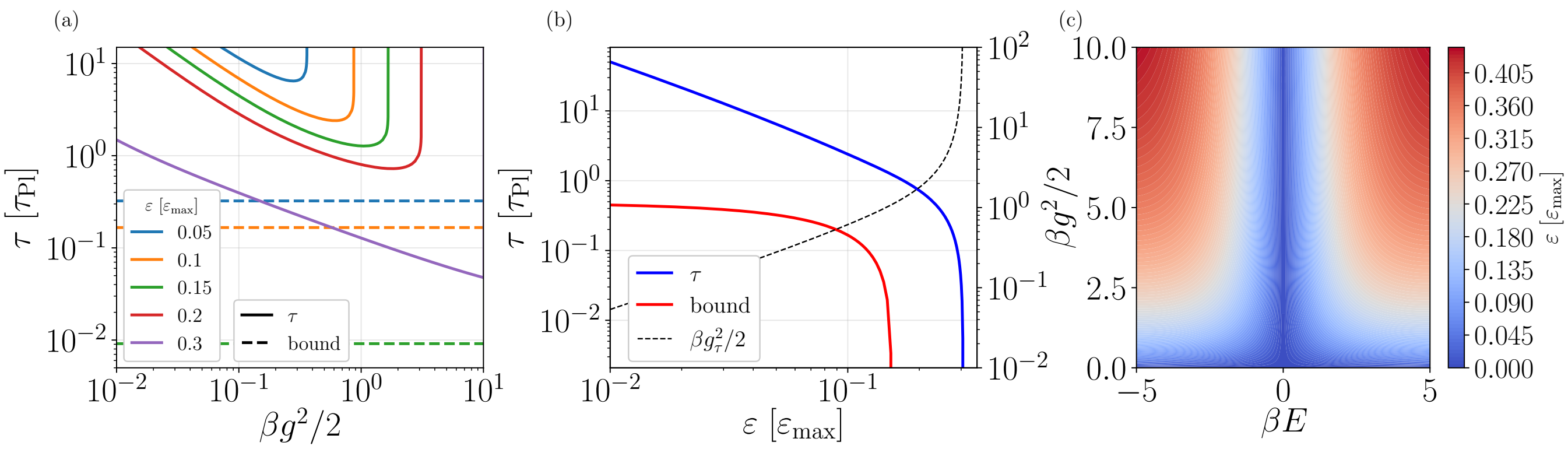

FIG. S5. **Thermalization machine with constant coupling.** Since for $p(0) = \frac{1}{2}$ and $E_S = 0$ this machine can instantly thermalize the system, Eq. (G.5) imposes a bound on the thermalization speed for $E_S \neq 0$. Here we fix $E_S^{(1)} = 0$ and $E_S^{(2)} = k_B T$ to illustrate the bound in different scenarios. By construction, for these energies, bound (G.5) becomes trivial for $\varepsilon \geq 0.153\varepsilon_{\max}$. **(a)** Minimal thermalization time τ (plain lines) of the machine as a function of the coupling g for multiple values of the accepted precision for thermalization. The dashed lines correspond to bound (G.5) of the corresponding ε, for $\varepsilon = 0.2\varepsilon_{\max}$ and $\varepsilon = 0.3\varepsilon_{\max}$ the bound is trivial (negative). Indeed we can see that the thermalizing machine is respecting the bound and that by reducing the precision of the machine we can achieve faster thermalization. At a given value of g and above the machine fails to thermalize (the required time blow up to infinity) as the global thermal state it is converging to is too far from the local thermal state. **(b)** Minimal thermalization time (blue) the machine can achieve by picking the optimal coupling as a function of the precision ε (minima of the curves in (a)). The corresponding coupling g_τ that achieves the thermalization time τ is depicted with the dashed black line,on the other y-axis. The bound (Eq. (G.5)) is depicted by the red line. We can see that at $\varepsilon \approx 0.153\varepsilon_{\max}$ the bound becomes trivial, and that at $\varepsilon \approx 0.306\varepsilon_{\max}$ the thermalization time of the machine drops to zero because the initial state is within the machine precision. As we approach this limit, the optimal coupling diverges since the output state of machines with larger and larger couplings falls within the desired precision of thermalization ($\lim_{g\to\infty} \lim_{t\to\infty} p(t) = \frac{1}{2}$). **(c)** The precision required (i.e. the smallest value of ε) such that $\lim_{t\to\infty} p(t)$ (Eq. (G.6)) is considered to be thermal, as a function of E and g.

where Ψ is the digamma function. This does not correspond to the desired thermal probability $f_\beta(E)$ (cf. Fig. S5c). Therefore, despite the fact that it might seem that we can thermalize the state as fast as we wish by increasing g, the machine will output a state that is further away from the desired one the faster it is. Since we allow for an error on the thermalizing machine, we can test the bound given by Eq. (G.5) for this protocol. By picking $E_S^{(1)} = 0$ and $p(0) = \frac{1}{2}$ we find that the system's total (i.e. $S + M$) and local (i.e. only S) thermal state coincide and that the system does not evolve, therefore $D(\rho_S^{(1)}(t), \omega_\beta(H_S^{(1)})) = 0$ for all $t \geq 0$, which implies that the bound of Eq. (G.5) is always true for $H_S^{(2)}$. We illustrate how this limits the performance of the machine to thermalize $H_S^{(2)} = k_B T \hat{a}^\dagger \hat{a}$ in Fig. S5.

Since the bound of Eq. (G.5) is for the machine to thermalize *all* of $\mathcal{H}$ within an error ε. We can turn the statement around into a speed-limit of evolution by taking $\varepsilon = D(\rho_S(t), \omega_\beta(H_S))$ and considering times smaller than the bound. There must be at least one of the Hamiltonians which has not managed to thermalize. Therefore we can re-arrange the bound to give a limit on the Bures distance between the state at time t, $\rho_S^{(i)}(\tau)$ (that evolves with $H_S^{(i)} + H_M + V_{SM}(t)$), and its corresponding thermal state $\omega_\beta(H_S^{(i)})$

$$D(\rho_S^{(i)}(t), \omega_\beta(H_S^{(i)})) \geq \frac{1}{2} D(\omega_\beta(H_S^{(1)}), \omega_\beta(H_S^{(2)})) - \frac{t}{4\tau_{\rm Pl}} \beta \| H_S^{(1)} - H_S^{(2)} \| \tag{G.7}$$

for at least one among $i = 1$ and $i = 2$. It is interesting to note that in this form of the bound the RSH only contains terms that depend on the system, while all the dependence on the thermalizing machine is on the LSH. We can see this bound in action in Fig. S5 for a machine with constant coupling, where in panels (a) and (b) we showcase the time necessary to reach $\varepsilon = D(\rho_S(t), \omega_\beta(H_S))$. These results show very clearly that by increasing the coupling g we do reduce the thermalization time-scale $2g^{-2}$, however as we do so the system thermalizes further and further away from the desired local thermal state. Therefore we can be as fast as we desire, however this comes at the cost of precision.

A way to combat the fact that the system thermalizes to the "wrong" state, is to change the coupling in time. Indeed, from Eq. (G.6) we can obtain $\lim_{g\to 0} \lim_{t\to\infty} p(t) = f_\beta(E)$, which is the desired thermal output. Therefore we

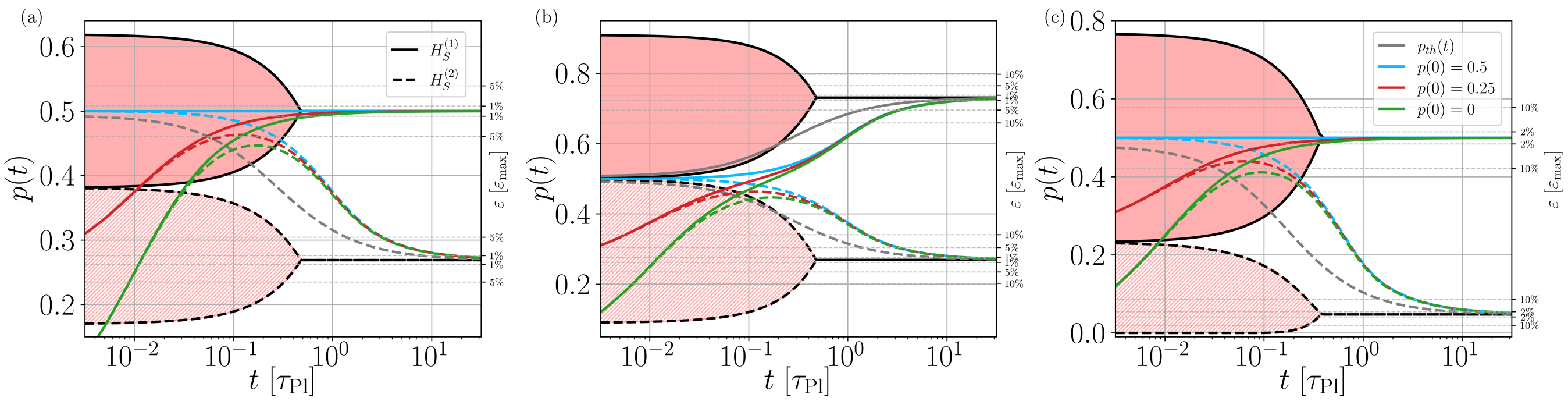

FIG. S6. **Thermalization machine with decaying coupling.** For two given Hamiltonians and the same initial condition, Eq. (G.7) defines a forbidden area of the state space $(p^{(1)}(t), p^{(2)}(t))$ as a function of time. Here it is illustrated with the red-shaded areas: if a given machine thermalizes quickly one Hamiltonian $H_S^{(1)}$ (the plain line enters the plain red area at time t) then it cannot thermalize a second Hamiltonian $H_S^{(2)}$ (the dashed line cannot enter the dashed red area at time t) with arbitrary precision in the same amount of time. This must hold for any conceivable M Hamiltonian that is able to thermalize S. Here, we illustrate for a machine with decaying coupling and $g(t) = \sqrt{a/(t+b)}$ for $a = \hbar$ and $b = \tau_{\rm Pl}/100$ and different choices of Hamiltonians: (a) $E_S^{(1)} = 0$, $E_S^{(2)} = k_B T$ (b) $E_S^{(1)} = -k_B T$, $E_S^{(2)} = k_B T$ (c) $E_S^{(1)} = 0$, $E_S^{(2)} = 3k_B T$. The plain lines correspond to the evolution with $H_S^{(1)}$ and the dashed lines correspond to the evolution with $H_S^{(2)}$. The second y-axis marks the distance to the thermal state in units of the maximal Bures angle $\varepsilon_{\rm max}$. The colors represents different initial conditions, while the gray lines represent the instantaneous thermal probability $p_{th}(t)$ (Eq. (G.6) with g replaced by $g(t)$). The instantaneous thermal state does not correspond to any evolution, as the actual state will always lag behind it and therefore sets a bound on the evolution of the system. We have a tradeoff here where in the limit of a small then the instantaneous thermal probability can violate the bound (as in (b), where a is small for the specific configuration of the other parameters), but the coupling is so weak that the actual probability of the system is far from it because it is thermalizing slowly since the coupling is weak. While when a is large we have that the instantaneous thermal state does not violate the bound (as in (a) and (c), where a is large for the specific configuration of the other parameters), and therefore the state won't be able to do so either.

want to consider machines where the coupling is a function of time such that $g(t)$ drops to zero smoothly. However, let us note that the first term of Eq. (G.4) is $p(0)e^{-\int_0^t dr g(r)^2}$, therefore if we pick a function $g(t)$ such that $\int_0^\infty dr g(r)^2 < \infty$ then we will have $e^{-\int_0^\infty dr g(r)^2} > 0$ implying that the system has not thermalized since the probability at $t = \infty$ is dependent on the initial probability $p(0)$. We can therefore conclude that for the system to thermalize exactly at $t = \infty$ the function $g(t)$ cannot decay faster than $t^{-1/2}$. Since we want the thermalization to happen as fast as possible, we study machines with functions of the form $g(t) = \sqrt{a/(t+b)}$. In this case we can simplify Eq. (G.4) to

$$p(t) = \frac{1}{2} + \left(p(0) - \frac{1}{2}\right)\left(\frac{b}{t+b}\right)^a - \frac{1}{2\beta}\frac{a}{(t+b)^a}\int_0^t ds ds'\ (s+b)^{\frac{a-1}{2}}(s'+b)^{\frac{a-1}{2}}\frac{\sin(E(s-s'))}{\sinh(\pi(s-s')/\beta)}\ . \tag{G.8}$$

From this we can compare the performance of this thermalizing machine to the bound given by Eq. (G.7), we look at a few examples and discuss them in Fig. S6.

ADDITIONAL REFERENCES

[i] Petz, D. (1996). *Monotone metrics on matrix spaces.* Linear algebra and its applications, 244, 81-96.
[ii] Nielsen, M. A., Chuang, I. L. (2010). *Quantum computation and quantum information.* Cambridge university press.
