# Supplementary material for: An information-theoretic proof of the Planckian bound for thermalization
Source: Nat Phys. 2026 Sep 24;22(11):1761–6. doi: 10.1038/s41567-026-03397-y (PMC13759322; doi:10.1038/s41567-026-03397-y)
Supplement: Supplementary file 2 [file 41567_2026_3397_MOESM2_ESM.pdf]

nature portfolio

Peer Review File

An information-theoretic proof of the Planckian bound for thermalization

Corresponding Author: Dr Paolo Abiuso

Version 0:

Reviewer comments:

Reviewer #1

(Remarks to the Author)
The authors present a rigorous, model-independent proof that quantum mechanics imposes a fundamental lower limit on how fast any system can thermalize. They show that the minimum possible relaxation time is of the order of the so-called “Planckian time,” which scales inversely with temperature and is determined solely by Planck’s and Boltzmann’s constants. At very low temperatures, this universal bound transitions to one governed by the system’s energy gap, linking the result to the quantum adiabatic theorem. The authors thus provide the first information-theoretic justification for the “Planckian bound” widely observed in experiments on strongly correlated and chaotic systems.

Their approach introduces the concept of a “thermalization machine”—an idealized quantum device that drives a system into thermal equilibrium under only two assumptions: that the evolution obeys quantum mechanics and that the device can produce thermal states for a range of Hamiltonians. Using tools from quantum information geometry and quantum metrology, the authors show that the distinguishability between the thermal states of different Hamiltonians sets a strict limit on how quickly any physical process can reach equilibrium.

They further analyze this limit through the lens of quantum Fisher information, revealing that it coincides with the ultimate precision bound allowed by the Heisenberg uncertainty principle. The paper also examines a simple fermionic model that approaches the theoretical limit, illustrating the practical meaning of the bound.

The bound itself was first inferred from empirical observations in the research area of superfluidity in 2004 in Ref. [12]. It is of high significance in condensed matter, quantum thermodynamics, and related fields.

While the bound has been derived in very limited settings (see Refs. [15, 16, 17]), their lack of universality greatly limits both the significance and applicability of the bound. The authors, on the other hand, provide the first universal proof of the bound: their result is highly general, as the assumptions going into it are minimal and well motivated. This is surprising, as it shows that this empirically observed bound possesses great universality. In addition, the authors show that their bound can be saturated in a very simple and physically meaningful example. In my view, these facts greatly elevate the importance of the result, putting it on par with the laws of thermodynamics or Carnot's universal efficiency bounds for heat engines.

One key property of their proof method is that it avoids making assumptions about the existence of a “light-cone bound” (i.e., a Lieb-Robinson bound). Related bounds in the literature often assume it exists, but it is well known not to hold in general [see e.g. Phys. Rev. Lett. 102, 240501 (2009)]. A case in point is the third law of thermodynamics, which can be phrased as: “Zero temperature cannot be reached in finite time.” If one tries to lower-bound the time required to reach absolute zero, their result implies that it cannot be achieved in finite time by any thermalization machine, thus implying the 3rd law in the above-stated form. Another paper that proves a form of the third law [Nature Communications 8, 14538 (2017)] assumes that Lieb-Robinson bounds hold universally, which limits its impact. This is just one of many such papers. It is very impressive how the authors use a quantum-speed-limit/metrology-inspired proof, rather than a Lieb-Robinson-inspired one, thereby circumventing this large assumption and achieving a far more general result.

Arguably, the most prominent assumption the authors make is that their thermalization machine functions independently of the system Hamiltonians it is required to thermalize (the ones in the delta-ball). The authors justify why this is an essential and reasonable assumption, by pointing out that removing it would allow instantaneous thermalization, which is unphysical. I also think it has a more physical justification: environments or “Thermalization Machines”—to use the authors’ language—that are capable of thermalizing a particular system should also be able to thermalize it if the system parameters change ever so slightly. Any system that doesn’t satisfy this principle would require artificial fine-tuning.

The paper is very well written and takes into account the intended audience of Nature Physics: the introduction is accessible to any scientist and explains the core message and results of the paper in a simple and easy-to-understand form. The later sections delve into details so that expert readers can appreciate the subtleties. The figures are clearly explained and the graphics well presented. The text does not exaggerate its claims, and the conclusions are well justified given the results. I have verified most of the proof in the supplementary material.

In conclusion, such powerful, universal, elegant, and tight bounds are rare in theoretical physics. In my opinion, these are all hallmarks of a great paper that will withstand the test of time. I fully recommend this paper for publication in Nature Physics.

--Small technical points:--

---In Eq. (5), I recommend adding more space before and after the symbol '|'. It took me a few seconds to parse the notation; a bit more spacing (which is conventional) would help. Alternatively, you could replace '|' with "such that," which is equivalent.

---You write: "Finally, it is worth mentioning that both conditions (i) and (ii) are chosen for simplicity and can be relaxed." Technically, (i) and (ii) in Requirement 2 form a single condition, not two separate ones, since they are linked by "for all." One option is to merge the labels (i) and (ii) into one and refer to the first and second equations as Eq. (4) and Eq. (5), respectively. (In some other places, you refer to them as separate conditions too.)

---In the text above Eq. (7), you write $\varepsilon/\beta \lll \delta \to 0$. I understand this to mean that you consider the limit $\delta \to 0$ under the constraint that $\varepsilon/(\delta\beta) \to 0$ as $\delta \to 0$. This seems very reasonable physically, but I would use more precise notation than the $\lll$ symbol, as its exact meaning can be ambiguous.

---On page 3: replace "can not" with "cannot."

---On page 2: the sentence "This machine should be able to thermalize the system S for at least some choices of H_S, which is illustrated in Fig. 1 and formalized as follows:" could be made clearer. I understand it to mean that the thermalization machine defined in "Machine Requirement 1" is a function of H_S. That is to say, V_{SM}(t) + H_M(t) is independent of all H_S in the δ-ball (defined in Eq. (5)). This language could be sharpened to make that crystal clear.

---On page 4 of the Supplementary: in Eq. (B.22), the \lceil and \rceil commands could be made a bit larger to fully encapsulate the fraction n/k.

---In the text below Supplementary Eq. (C.11), change ".It then" to ". It then" (missing space).

---On page 7 of the Supplementary, second line: there is a missing right bracket in the expression for D(.).

Reviewer #2

(Remarks to the Author)
"An information-theoretic proof of the Planckian bound for thermalization" by Abiuso et al, proves a tighter bound on the thermalization time than the Planckian dissipation time in quantum systems by using information theoretic and information geometric tools. The work starts by defining two assumptions, R1 assuming quantum mechanics and R2 assuming thermalization happens where thermalization is defined as an initial state attaining a Gibbs state after time evolution. The main result lies on the sensible observation that distinguishability between two Gibbs states generated by two different system Hamiltonians is fundamentally constrained by the sensitivity of the unitary time evolution to changes in the system Hamiltonian. Tools like Bures distance, triangle inequality, quantum fisher information are utilized in proofs.

The work provides a model-independent lower bound that ties thermalization time to Planckian time. This connection looks clean and broadly framed, i.e., independent of a particular microscopic bath model. Particularly they try to answer "can one thermalize a ball of Hamiltonians in the Hilbert space within an epsilon accuracy?" While I appreciate that stripping the problem from its physical constraints, and stating it instead operationally and as a mathematical problem helps authors prove a generic (model independent) bound, I don't find the result physically and practically useful. One main criticism I have is that the authors do not discuss the effect of bath-system interaction anywhere in their work. Authors state how their assumptions are the minimal assumptions for this bound to work. However for thermalization to happen in the first place, bath-system interaction has to be chosen such that energy can be exchanged between two parts. How is then there is no restriction on the interaction Hamiltonian?
Clearly the choice of interaction Hamiltonian will affect the thermalization time. Not discussing this at all I think severs the connection of the work to many-body physics and condensed matter.

Additionally, it would help to clarify how the bound relates to the cases where the system can only achieve generalized gibbs ensemble such as in integrable or MBL systems. In other words, one has to specify the domain of H_S for which R2 is intended.

Thermalization and its timescales as well as correlation spreading across quantum many-body systems are affected by the locality of the underlying system, e.g., Lieb-Robinson bounds. How does the derived bound relate to Lieb-Robinson bounds?

How would the result change if one uses other distance metrics different than Bures, such as trace distance? Trace distance is experimentally motivated.

Another main criticism I have is the introduction of the concept "thermalization machine". The authors seem to be re-inventing the wheel by introducing a new name for "bath" or "environment", which they call "thermalization machine". I don't see the point of this re-branding. What this "machine" does is to thermalize, which is exactly what's called in physics a bath. Additionally calling a bath a "machine" gives the impression that thermalization is an active process but thermalization is actually passive, there is no work involved in it. A machine however requires work to operate. I found this re-branding of bath very confusing and unnecessary.

Some key literature is missing:
First of all "ETH" is not written explicitly anywhere before using its abbreviation. Secondly, ETH is eigenstate thermalization hypothesis. So one does not need to repeat hypothesis after ETH again: ETH hypothesis is written before Eq. 2. Thirdly, early ETH literature is not cited, such as Srednicki's Phys. Rev. E 50, 888 (1994), Deutsch's Phys. Rev. A 43, 2046 (1991), M. Rigol, V. Dunjko, and M. Olshanii, Nature (London) 452, 854 (2008) etc.
The quantum speed limits literature gives lower bounds on the time for achieving a particular state under non-unitary maps, which is then relevant to the current work. For example: del Campo et al., Phys. Rev. Lett. 110, 050403 (2013), Deffner & Lutz, Phys. Rev. Lett. 111, 010402 (2013).

Some minor points: In Fig. 1 the authors state "this should happen to an error eps and for a nontrivial subset of hamiltonians". What defines "nontrivial subset of hamiltonians"? It might be useful to spell this out maybe in a footnote.
Before R1, the authors say "In order to minimize τ , we allow any control on M and its interaction with S, though they should —to some extent—be independent of the specifics of S."
This sounds very vague. What determines "some extent"? Can you elaborate?
Before Section B: Authors say "omega is sufficiently mixed" do they mean the system is almost at infinite temperature?
Same paragraph: they say "we derive a different bound" but I think here they can be more precise: "we derive a tighter bound". Since the previous bound in this limit p -> 1 gives chi >= 0, but the expression with the gap actually gives a tighter bound than this.

Overall, I think the idea behind the work is interesting (proving a universal bound) but its physics is abstracted away. I cannot see how the impact of the work can exceed its home community (information theory) to the neighbor communities like quantum many-body physics, condensed matter theory and AMO physics. Hence in my opinion the work currently does not meet the Nature Physics bar. Are there physical models or materials that can saturate this new tighter bound? Alternatively, perhaps the new bound can rule out the fast thermalization of a model that is widely believed to saturate bounds in many-body community, e.g., SYK model? Discussion of a compelling path to experiment or large-scale numerics would be very helpful. In conclusion, this work as a clean and generic information theoretic proof of a tighter bound on thermalization deserves publication in a more specialized journal, such as one on quantum information theory.

Version 1:

Reviewer comments:

Reviewer #2

(Remarks to the Author)
The authors made substantial revisions to their manuscript clarifying previously confusing points and extending the applicability of their theory. I recommend for its publication.

Open Access This Peer Review File is licensed under a Creative Commons Attribution 4.0 International License, which permits use, sharing, adaptation, distribution and reproduction in any medium or format, as long as you give appropriate credit to the original author(s) and the source, provide a link to the Creative Commons license, and indicate if changes were made.
In cases where reviewers are anonymous, credit should be given to 'Anonymous Referee' and the source.
The images or other third party material in this Peer Review File are included in the article's Creative Commons license, unless indicated otherwise in a credit line to the material. If material is not included in the article's Creative Commons license and your intended use is not permitted by statutory regulation or exceeds the permitted use, you will need to obtain permission directly from the copyright holder.
To view a copy of this license, visit https://creativecommons.org/licenses/by/4.0/

We thank both Referees for their detailed assessment of our work. We thank Referee 1 for the highly positive review as well as the technical comments which we address below. Referee 2 presents a more critical point of view, concerned in particular with the information-theoretic stance taken by the paper and the possible lack of physical applications of our results. We argue below why our minimalistic approach represents a strength and not a weakness of our results. Furthermore, we address technical points and provide additional analysis (including a new section in the manuscript with a connection to the Lieb-Robinson bound) regarding applications of our results.

In the following, the response in detail.

Referee 1

The authors present a rigorous, model-independent proof that quantum mechanics imposes a fundamental lower limit on how fast any system can thermalize [...] The authors thus provide the first information-theoretic justification for the "Planckian bound" widely observed in experiments on strongly correlated and chaotic systems. [...] The bound itself was first inferred from empirical observations in the research area of superfluidity in 2004 in Ref. [12]. It is of high significance in condensed matter, quantum thermodynamics, and related fields.

We thank the Referee for appreciating the relevance of our work.

While the bound has been derived in very limited settings (see Refs. [15, 16, 17]), their lack of universality greatly limits both the significance and applicability of the bound. The authors, on the other hand, provide the first universal proof of the bound: their result is highly general, as the assumptions going into it are minimal and well motivated. This is surprising, as it shows that this empirically observed bound possesses great universality. In addition, the authors show that their bound can be saturated in a very simple and physically meaningful example. In my view, these facts greatly elevate the importance of the result, putting it on par with the laws of thermodynamics or Carnot's universal efficiency bounds for heat engines.

We thank the Referee for appreciating the generality of our proof and its significance as an ultimate limit of thermodynamics within quantum theory.

One key property of their proof method is that it avoids making assumptions about the existence of a "light-cone bound" (i.e., a Lieb-Robinson bound). Related bounds in the literature often assume it exists, but it is well known not to hold in general [see e.g. Phys. Rev. Lett. 102, 240501 (2009)]. A case in point is the third law of thermodynamics, which can be phrased as: "Zero temperature cannot be reached in finite time." If one tries to lower-bound the time required to reach absolute zero, their result implies that it cannot be achieved in finite time by any thermalization machine, thus implying the 3rd law in the above-stated form.

We thank the Referee for this comment. The third law has been subject to intensive debate since the early 1900s, reaching a consensus (regarding its exact formalization) that is somewhat less canonical than for the 0th, 1st and 2nd law. Moreover, in the limits of $T \to 0$ the validity of our main bound $\tau \geq \beta\hbar/2$ requires accessing systems with smaller and smaller gaps, i.e. $\beta\Delta \nrightarrow \infty$, as to remain sufficiently mixed while the temperature decreases. In fact, as we write in the text, in such limit the meaningful bound becomes the adiabatic $\tau \geq \hbar/\Delta$. For these reasons, we do not make extensive claims about the third law of thermodynamics in the paper, whereas in the new version of the manuscript we comment at the end how our results might provide new insight on it.

Another paper that proves a form of the third law [Nature Communications 8, 14538 (2017)] assumes that Lieb-Robinson bounds hold universally, which limits its impact. This is just one of many such papers. It is very impressive how the authors use a quantum-speed-limit/metrology-inspired proof, rather than a Lieb-Robinson-inspired one, thereby circumventing this large assumption and achieving a far more general result.

Arguably, the most prominent assumption the authors make is that their thermalization machine functions independently of the system Hamiltonians it is required to thermalize (the ones in the delta-ball). The authors justify why this is an essential and reasonable assumption, by

pointing out that removing it would allow instantaneous thermalization, which is unphysical. I also think it has a more physical justification: environments or "Thermalization Machines"—to use the authors' language—that are capable of thermalizing a particular system should also be able to thermalize it if the system parameters change ever so slightly. Any system that doesn't satisfy this principle would require artificial fine-tuning.

We thank the Referee for the comment. In the new version they might appreciate we made an additional effort in stressing this point, clarify its role and formalization (see e.g. changes in blue after R2 in the first page, and between Requirement 1 and Requirement 2 in the second page).

The paper is very well written and takes into account the intended audience of Nature Physics [...]. In conclusion, such powerful, universal, elegant, and tight bounds are rare in theoretical physics. In my opinion, these are all hallmarks of a great paper that will withstand the test of time. I fully recommend this paper for publication in Nature Physics.

Once again, we thank the Referee for their assessment.

–Small technical points:–

—In Eq. (5), I recommend adding more space before and after the symbol '|'. It took me a few seconds to parse the notation; a bit more spacing (which is conventional) would help. Alternatively, you could replace '|' with "such that," which is equivalent.

We added a space to improve readability as suggested.

—You write: "Finally, it is worth mentioning that both conditions (i) and (ii) are chosen for simplicity and can be relaxed." Technically, (i) and (ii) in Requirement 2 form a single condition, not two separate ones, since they are linked by "for all." One option is to merge the labels (i) and (ii) into one and refer to the first and second equations as Eq. (4) and Eq. (5), respectively. (In some other places, you refer to them as separate conditions too.)

We thank the Referee for the attentive revision. we now refer to (i) and (ii) in Requirement 2 without calling them "conditions".

—In the text above Eq. (7), you write $\varepsilon/\beta \ll \delta \to 0$. I understand this to mean that you consider the limit $\delta \to 0$ under the constraint that $\varepsilon/(\delta\beta) \to 0$ as $\delta \to 0$. This seems very reasonable physically, but I would use more precise notation than the $\ll$ symbol, as its exact meaning can be ambiguous.

We modified the text introducing Eq.(7) in order to clarify the meaning that was correctly pointed out by the Referee, namely that the limit is taken on δ while keeping the ratio $\varepsilon/(\delta\beta)$ negligible. Formally, this is equivalent to taking first the limit on ε and then on δ. In the equation itself we kept the notation $\varepsilon/\beta \ll \delta \to 0$ which compactly signifies the condition $\varepsilon \ll \beta\delta$ necessary to neglect the error term in Eq.(6), as well as the fact that the limit is taken on the size δ of the ball (i.e. β is fixed).

—On page 3: replace "can not" with "cannot."

Replaced as suggested.

—On page 2: the sentence "This machine should be able to thermalize the system S for at least some choices of H_S, which is illustrated in Fig. 1 and formalized as follows:" could be made clearer. I understand it to mean that the thermalization machine defined in "Machine Requirement 1" is a function of H_S. That is to say, $V_{SM}(t) + H_M(t)$ is independent of all H_S in the δ-ball (defined in Eq. (5)). This language could be sharpened to make that crystal clear.

The Referee is correct. We modified the text introducing Requirement 2 into a more precise wording as "In order to represent a nontrivial thermalization process, such a machine should thermalize *S for multiple choices of H_S* via a *fixed routine* $\{H_M(t), V_{SM}(t)\}$.".

—On page 4 of the Supplementary: in Eq. (B.22), the \lceil and \rceil commands could be made a bit larger to fully encapsulate the fraction n/k.

—In the text below Supplementary Eq. (C.11), change ".It then" to ". It then" (missing space).

—On page 7 of the Supplementary, second line: there is a missing right bracket in the expression for D(.).

All typos corrected as suggested.

Referee 2

An information-theoretic proof of the Planckian bound for thermalization" by Abiuso et al, proves a tighter bound on the thermalization time than the Planckian dissipation time in quantum systems by using information theoretic and information geometric tools. [...] The work provides a model-independent lower bound that ties thermalization time to Planckian time. This connection looks clean and broadly framed, i.e., independent of a particular microscopic bath model. [...] While I appreciate that stripping the problem from its physical constraints, and stating it instead operationally and as a mathematical problem helps authors prove a generic (model independent) bound, I don't find the result physically and practically useful.

We thank the Referee for appreciating the generality of our result. Indeed this is the main message of our paper: a general proof of a fundamental bound dictated by quantum mechanics. Similarly to other results on fundamental physics, the scales involved might be, as expected, out of reach from current technologies and without immediate practical use. However, we believe this does not necessarily strip our results from its significance (e.g. current computers operate orders of magnitude above Landauer's limit, but this does not diminish its fundamental importance). Our work addresses a long-standing question in condensed matter and related fields, where there has been sustained debate across different approaches regarding the existence and nature of a fundamental timescale for thermalization. Our result formalizes this notion and establishes a universal and tight lower bound, thereby adding a crucial insight into an important open question in physics, as also noted by Referee 1.

To reach a universal result, a central conceptual contribution of our work is to take seriously a feature of thermalization that is often implicitly assumed: robustness with respect to variations in the system. Namely, thermalization should be a process that drives a system governed by a Hamiltonian H to its corresponding Gibbs state $\rho \propto e^{-\beta H}$. Crucially this requirement should hold not just for a single fixed Hamiltonian, but for a family of system Hamiltonians. By imposing this consistency requirement, while otherwise allowing the thermalization process to be as general as quantum mechanics permits, we show that a fundamental lower bound on the thermalization time necessarily emerges.

While this operational approach may appear to disconnect our results to physical systems of interest (e.g., many-body or condensed-matter settings), we emphasize that this is not the case. To address this concern, in the current version we explicitly show that our work introduces a general framework and a set of tools that can be used to derive tighter bounds in concrete scenarios of physical relevance. In response to the Referee's comments, we have added a new section (Sec.III C) explaining how this framework applies to scenarios involving partial observables, non-thermal systems, and short-range interacting systems (thus allowing a connection to Lieb–Robinson bounds).

One main criticism I have is that the authors do not discuss the effect of bath-system interaction anywhere in their work. Authors state how their assumptions are the minimal assumptions for this bound to work. However for thermalization to happen in the first place, bath-system interaction has to be chosen such that energy can be exchanged between two parts. How is then there is no restriction on the interaction Hamiltonian? Clearly the choice of interaction Hamiltonian will affect the thermalization time. Not discussing this at all I think severs the connection of the work to many-body physics and condensed matter.

We thank the Referee for their comments and we respectfully disagree with the statement that this is a negative aspect of our work. As explained above, a crucial contribution of our work is that a fundamental lower bound arises from a single restriction on robustness of thermalization. We see the lack of further assumptions as a *major strength* of our result: if we were to impose more contraints, the bound would become less general and depend on more physical properties. There are very seldom non-trivial statements in theoretical physics that are so universal (e.g. Carnot's or Landauer bound). For example, Referee 1 points out *"While the bound has been derived in very limited settings (see Refs. [15, 16, 17]), their lack of universality greatly limits both the significance and applicability of the bound. The authors, on the other hand, provide the first universal proof of the bound".*

We wish to further clarify an important conceptual point underlying our approach. While it

is certainly true that specific choices of the system–bath interaction Hamiltonian determine the actual thermalization dynamics and therefore the thermalization time in concrete models, this is not in contradiction with our result. Rather, our work addresses a different question: what is the minimum thermalization time allowed by quantum mechanics, independently of microscopic details. At first sight, one might be tempted to think that in the absence of constraints on the interaction Hamiltonian there is no nontrivial lower bound on thermalization time. Our main result precisely shows that this intuition is incorrect. We prove that quantum mechanics imposes a rigid, model-independent lower bound on the time required for thermalization, which depends only universal fundamental constants, namely Boltzmann's constant, setting the scale of statistical physics, and Planck's constant, setting the scale of quantum mechanics; and not on the detailed structure of the interaction.

Importantly, our result should not be interpreted as a statement about typical thermalization times in realistic many-body or condensed-matter systems. Instead, it identifies a fundamental speed limit: no matter how one engineers the bath or the interaction Hamiltonian, if a single thermalization process is capable of thermalizing a system for a set of distinct system Hamiltonians, then the thermalization time cannot be shorter than the bound we derive. Specific interactions may lead to much slower dynamics (or no thermalization at all), but never to faster ones. From this perspective, the absence of assumptions on the system–bath interaction is not a disconnect from many-body physics, but rather what allows us to isolate the purely quantum–thermodynamic origin of the bound. Once additional physical structure is imposed (e.g., locality, weak coupling, spectral properties of the bath, dimensionality, or transport constraints typical of condensed-matter systems), one indeed expects tighter but less universal bounds. We fully agree with the referee that understanding such refined bounds is an important direction for future research. The newly added Sec.III C is meant precisely as a guide for how our general framework can be specialized to concrete physical scenarios. It provides a prescription for incorporating additional assumptions on the interaction in order to derive sharper bounds tailored to specific classes of systems, without obscuring the fundamental and universal nature of the present result.

These arguments are reflected in the new version of the manuscript, where we improved the clarity of these points (see modifications in blue, both in the introduction and the conclusions), and further stressed the generality of proof and its applications.

Additionally, it would help to clarify how the bound relates to the cases where the system can only achieve generalized gibbs ensemble such as in integrable or MBL systems. In other words, one has to specify the domain of H_S for which R2 is intended.

We thank the referee for the question, as it brings up a very interesting point. The bound we derived can be very directly adapted to states that are not described by Gibbs distributions. In the old version of the manuscript this was only very briefly alluded to in the discussion. This is one of the main points of the new section we have added to the manuscript.

In short, there are multiple versions of our bound that can be obtained based on three choices: *a)* the class of target states/distributions that the machine should output (e.g. thermal, reduced thermal state, generalized Gibbs ensemble, MBL steady states, etc.); *b)* constraints on how the distribution is witnessed, i.e. does the observer have access to the full state (e.g. with tomography), or only via a set of observables (e.g. measuring energy, momenta, or local magnetization); *c)* constraints on the machine (e.g. is it a thermal bath or a quantum computer? Can it generate entanglement in the system?). With these "levers" one can apply the same technique to obtain different versions of our bound, that either apply in different scenarios (e.g. generalized Gibbs ensembles) or more specific scenarios (e.g. only passive thermal environments for machines).

In the new Sec.III C we explain this procedure in detail and provide an inequality (Eq.(8)) that can be readily applied to different scenarios by changing these assumptions. As an example, we include *i)* a compact discussion on how critical scaling close to a phase transition or the spread of entanglement allowed in the machine affect the asymptotic scaling of our bound with the system's size, and *ii)* a specific application to a closed system scenario in which an initially uncorrelated system, subject to short-ranged interactions and that only thermalizes locally, in the spirit of the ETH.

Thermalization and its timescales as well as correlation spreading across quantum many-body systems are affected by the locality of the underlying system, e.g., Lieb-Robinson bounds. How

does the derived bound relate to Lieb-Robinson bounds?

We thank the Referee for the relevant question, which has motivated us to explore further the applicability of our framework. First, as extensively discussed above, our bound holds regardless of the specific topology of the interactions. This makes our approach quite unique: in words of the first Referee: *"It is very impressive how the authors use a quantum-speed-limit/metrology-inspired proof, rather than a Lieb-Robinson-inspired one, thereby circumventing this large assumption and achieving a far more general result."*

Now, as Referee 2 suggests, an interesting question is how our framework can be accommodated to account for physically well-motivated Hamiltonians, namely local and short-range interactions. When making such assumptions, the Lieb-Robinson bound imposes a stricter bound on the growth of the dynamical Quantum Fisher Information beyond the standard Heisenberg limit (see the recent work arxiv.2511.14855) –assuming the initial state to be uncorrelated. Exploiting our approach, this immediately translates into a *tighter* constraint on the thermalization time, see Eq. (10) in the new version of the paper. This makes a natural link between our framework and Lieb-Robinson bound.

How would the result change if one uses other distance metrics different than Bures, such as trace distance? Trace distance is experimentally motivated.

Similarly to the trace distance, the Bures angle is also operationally motivated as it is defined from the fidelity: $D_A(\rho,\sigma) = \arccos\sqrt{F(\rho,\sigma)}$ (and the Bures distance as $D_B(\rho,\sigma) = 2(1-\sqrt{F(\rho,\sigma)})$), and as such is used as a canonical distinguishability quantifier in numerous applications. Furthermore, the Bures distance and angle are experimentally relevant because they are related to the Fisher information, i.e. the metrological sensitivity of a state to a parameter; via the Cramér–Rao inequality this sensitivity yields an experimental bound that can be saturated asymptotically.

Mathematically speaking, the Bures distance and Bures angle are topologically equivalent to the trace distance, as they are mutually constrained via the Fuchs–van de Graaf inequality, which can be written as

$$1-\cos(D_A(\rho,\sigma)) \leq \frac{||\rho-\sigma||_1}{2} \leq \sin(D_A(\sigma,\rho)) \ . \tag{1}$$

For these reasons we stick to the choice of Bures angle as a quantifier of distance in our work, which naturally appears in the derivations and is operationally relevant.

For the sake of completeness, let us show that similar bounds can be derived for the case of the trace distance. Namely consider our main inequality

$$\tau \geq \tau_{\mathrm{Pl}} \cdot \left[\frac{2D_A\big(\omega(\beta,H_S^{(1)}),\omega(\beta,H_S^{(2)})\big)-4\varepsilon}{\beta\,\|H_S^{(1)}-H_S^{(2)}\|}\right] \ , \tag{2}$$

it follows immediately from the F-vdG inequality (Eq. (1)) that this implies

$$\tau \geq \tau_{\mathrm{Pl}} \cdot \left[\frac{||\omega(\beta,H_S^{(1)})-\omega(\beta,H_S^{(2)})||_1-4\varepsilon}{\beta\,\|H_S^{(1)}-H_S^{(2)}\|}\right] \ , \tag{3}$$

therefore an equivalent inequality holds for the trace distance. This bound will clearly provide different numerical values (smaller in general), however let us notice that qualitatively it will have a similar behavior: as an example, for sufficiently mixed states such that an approximate 50%-50% bipartition can be found, i.e.

$$\omega = \sum_i p_i\,|i\rangle\!\langle i| \quad \text{and} \quad \exists\ \mathcal{I} \ \Big| \ \sum_{i\in\mathcal{I}} p_i \approx \frac{1}{2} \ , \tag{4}$$

we obtain lowerbounds to χ by considering variations of the form

$$\delta\omega = \alpha\left(\sum_{i\in\mathcal{I}}|i\rangle\!\langle i| - \sum_{i\notin\mathcal{I}}|i\rangle\!\langle i|\right) \tag{5}$$

(cf. manuscript). For $\sum_{i\in\mathcal{I}} p_i \approx \frac{1}{2}$ this is translated locally to

$$||\delta\omega||_1 = 2\alpha \approx 2D_A(\omega, \omega + \delta\omega) \tag{6}$$

at leading order. Thus the lowerbound on χ for sufficiently mixed states would also read $\chi \gtrsim 0.5$ in a trace-distance formulation of the same work.

Another main criticism I have is the introduction of the concept "thermalization machine". The authors seem to be re-inventing the wheel by introducing a new name for "bath" or "environment", which they call "thermalization machine". I don't see the point of this re-branding. What this "machine" does is to thermalize, which is exactly what's called in physics a bath. Additionally calling a bath a "machine" gives the impression that thermalization is an active process but thermalization is actually passive, there is no work involved in it. A machine however requires work to operate. I found this re-branding of bath very confusing and unnecessary.

We thank the Referee for the comment as it brings up a crucial point. While the concept of thermalization machine *includes* thermal baths, it is not limited to it. We explicitly allow for active processes (indeed we allow the machine and interaction Hamiltonians to be time-dependent). More specifically, our general bound includes (passive) thermal environments but also (active) cooling/thermalization processes, even highly engineered cooling machines (that would presumably require a high amount of work). Indeed, there are multiple active and operational contexts (e.g. Quantum Control experiments in AMO, optomechanics, superconducting systems, etc.) where cooling (or more generally Gibbs states preparation) is crucial, and therefore could be done by an active process. What we show is that even active processes are subject to the Planckian thermalization bound, regardless of how engineered it is or how much work it requires (again, the crucial requirement is Requirement 2, namely the machine should be able to thermalise more than one specific system).

Furthermore, in section IV (formerly III.C) we saturate the bound with a machine that uses Hamiltonian discrimination, this is definitely not just a bath but a complex active process (that could be realized by e.g. a quantum computer). Let us also underline that this does not mean that we require an active process: we simply allow for it. For example, in section IV.A (formerly III.D) the machine is a thermal bath: in the main text the process is active (via control of the interaction), but in the supplemental material we also cover the same case with a passive process where the interaction is kept constant.

While we certainly do not aim to create confusion with this nomenclature, we believe it is important to emphasize that our bound applies to both passive and active cooling/thermalization processes. *Thermalizing machine* is the best name we could think of, but we are surely open to suggestions. In any case, we have changed the text of the manuscript in the appropriate places to clearly highlight this point and avoid further misunderstandings.

Some key literature is missing: First of all "ETH" is not written explicitly anywhere before using its abbreviation. Secondly, ETH is eigenstate thermalization hypothesis. So one does not need to repeat hypothesis after ETH again: ETH hypothesis is written before Eq. 2. Thirdly, early ETH literature is not cited, such as Srednicki's Phys. Rev. E 50, 888 (1994), Deutsch's Phys. Rev. A 43, 2046 (1991), M. Rigol, V. Dunjko, and M. Olshanii, Nature (London) 452, 854 (2008) etc. The quantum speed limits literature gives lower bounds on the time for achieving a particular state under non-unitary maps, which is then relevant to the current work. For example: del Campo et al., Phys. Rev. Lett. 110, 050403 (2013), Deffner & Lutz, Phys. Rev. Lett. 111, 010402 (2013).

We changed the wording referring to ETH, by making it explicit, and we do not introduce its acronym, as it only appears once in the manuscript. We also thank the Referee for the suggested references:

- On the topic of ETH, we have opted to retain our selection of comprehensive reviews—one primarily pedagogical and the other more technical, both featuring contributions from the authors of the seminal works—rather than adding the individual foundational ETH papers. We also added the M. Rigol et al., Nature (2008) paper suggested by the Referee, as it also provides a seminal reference in modern language suitable to the general audience. It is also important to notice that methodologically and conceptually, our approach is distinct from

the ETH framework. We believe that directing the reader to these reviews provides the most effective context without misleadingly suggesting that our derivations are a direct extension of the ETH formalism.

- Regarding speed limits, as we clarify in the manuscript, these cannot be used a priori to derive useful bounds for our purposes, as they require additional assumptions about the type of interactions induced by the thermalization machine —assumptions that we intentionally avoid making. The link between speed limits and our technique is however relevant, both from the point of view of the similar mathematics, as well as because it is useful put them contrast with our results, for the reasons above. We therefore added the two references suggested by the Referee, together with the one we previously included (now forming Ref.s[24-26]), in the introduction of the paper, when depicting the idea behind our framework and the difference between state preparation and thermalization machines.

Finally, we would like to mention that we have been selective in the choice of references in order to try to adhere to the small number of references prescribed by Nature Physics.

Some minor points: In Fig. 1 the authors state "this should happen to an error eps and for a nontrivial subset of hamiltonians". What defines "nontrivial subset of hamiltonians"? It might be useful to spell this out maybe in a footnote.

A nontrivial set of Hamiltonian is intended to be a set with more than 1 element. We removed the old text and clarified the caption with "[...] for multiple choices of H_S (here forming a ball of size δ for simplicity)."

Before R1, the authors say "In order to minimize τ, we allow any control on M and its interaction with S, though they should—to some extent—be independent of the specifics of S." This sounds very vague. What determines "some extent"? Can you elaborate?

We agree with the Referee that the previous wording was vague and unnecessary. In the new version we modified it to "they should be independent of the specifics of S alone", which explains in text what is formalized just after, namely that the same protocol $\{V_{SM}(t), H_M(t)\}$ should work for different values of H_S (see modified text in blue).

Before Section B: Authors say "omega is sufficiently mixed" do they mean the system is almost at infinite temperature?

The meaning of "sufficiently mixed" is clarified by the following "so that $p \approx \frac{1}{2}$ can be chosen". This means we do not need the temperature to be high: a thermal states featuring populations with values $\{0.5, 0.3, 0.2\}$ is sufficiently mixed as it features a trivial 50%-50% coarse-graining. Mathematically this is outlined as in the reply above (Eq. (4)). More generally, it is sufficient that the ground state probability is not "too big": in the manuscript we make, for simplicity, the example in which $p_0 \leq 2/3$, however full numerics can be appreciated in Fig.2.

Same paragraph: they say "we derive a different bound" but I think here they can be more precise: "we derive a tighter bound". Since the previous bound in this limit $p \to 1$ gives $\chi \geq 0$, but the expression with the gap actually gives a tighter bound than this.

We clarified this in the new version: this is a different bound to χ, which is tighter only in the high-p region.

Overall, I think the idea behind the work is interesting (proving a universal bound) but its physics is abstracted away. I cannot see how the impact of the work can exceed its home community (information theory) to the neighbor communities like quantum many-body physics, condensed matter theory and AMO physics. Hence in my opinion the work currently does not meet the Nature Physics bar. Are there physical models or materials that can saturate this new tighter bound? Alternatively, perhaps the new bound can rule out the fast thermalization of a model that is widely believed to saturate bounds in many-body community, e.g., SYK model? Discussion of a compelling path to experiment or large-scale numerics would be very helpful. In conclusion, this work as a clean and generic information theoretic proof of a tighter bound on thermalization deserves publication in a more specialized journal, such as one on quantum information theory.

We thank the referee for recognizing the universal character of our result. We respectfully disagree, however, with the assessment that this universality renders the result detached from

physical reality, or of interest primarily to a niche quantum-information audience. On the contrary, we believe that our contribution is well aligned with the type of work traditionally valued by Nature Physics: a simple and transparent no-go statement that reveals a highly nontrivial constraint on nature, involves only universal constants from quantum and statistical physics, and can be understood at face value, without requiring strong technical background in theoretical physics, mathematics, or computation. Beyond the result itself, the bound is supported by an unusually concise and elegant proof, and by a framework that introduces genuinely new methods. These methods are not only of conceptual interest, but can be directly adapted to address more specific models, inform large-scale numerical studies, and suggest concrete experimental protocols. We believe that this combination of conceptual clarity, universality, and methodological novelty places our work squarely within the scope and spirit of a broad-interest journal such as Nature Physics.

In this spirit, we note that the question of fundamental limits on thermalization times has been extensively debated in the condensed-matter and many-body communities, as reflected in existing reviews (see Refs. [5,6]). As pointed out by Referee 1, *"it is of high significance in condensed matter, quantum thermodynamics, and related fields"*.

To further substantiate the breadth of relevance, and to address potential connections highlighted by the referee, we briefly summarize some concrete links to different research communities:

- Condensed matter and Lieb-Robinson (LR) bounds: our framework can incorporate physically motivated constraints such as locality and short-range interactions, leading to tighter bounds on thermalization times (see Eq.(10)) that are directly connected to Lieb–Robinson bounds.

- Large-scale numerics: A key technical ingredient of our work is the comparison between static distinguishability measures (such as Bures or trace distance and quantum Fisher information) and dynamical distinguishability, which is closely linked to entanglement generation. Efficient numerical tools (most notably tensor-network methods) can be used to compute these quantities and derive sharper, model-specific bounds. This perspective is now clearly laid out in Sec. IIIC of the revised manuscript, with the quantities of interest appearing in Eq.s (8) and (9).

- Open quantum systems: Our theoretical results are tested against a paradigmatic model in non-Markovian open quantum systems and quantum transport, namely the fermionic resonant level model (see Sec. IV A). This provides a clear specific example of the applicability of our results to relevant thermalization models. Furthermore, it is worth stressing that the bound on thermalization time (shown as red regions in Fig. 3) apply to *any* thermalization model for a single qubit (e.g. the common spin-boson model, but also more complex ones).

- Experiments: The resonant level model described above is a standard model to describe thermalization in mesoscopic electronic devices, such as quantum dots or a metallic islands connected to conducting electron reservoirs. Both the dot's energy level and its coupling to the reservoir can be independently and dynamically tuned by applying appropriate gate voltages (see e.g. Phys.Rev.B **111**, 115117). This provides a natural path to experimentally testing the theoretical optimal thermalization protocols described in Sec. IV A.

To wrap up, let us reinstate the importance of fundamental bounds in physics, and highlight how our result contributes to this tradition. While it is correct that most physical systems thermalize on timescales much longer than the bound we derive, this does not diminish its conceptual significance. At the risk of repeating ourselves, we emphasize that universal lower bounds are not intended to describe typical behavior in specific models, but rather to identify fundamental constraints imposed by the underlying physical principles. Prior to our work, no general argument excluded the possibility of arbitrarily fast—or even instantaneous—thermalization on purely quantum-mechanical grounds. Our result shows that quantum mechanics and thermodynamics together enforce a nonzero minimal timescale, $t \geq \beta\hbar/2$, independently of microscopic details or the form of the system–bath interaction. In this sense, our bound can be viewed as a modest addition to the same conceptual family as other foundational constraints and no-go results in physics, such as the Carnot bound on thermodynamic efficiency, the Landauer bound on the energetic cost of information erasure, quantum speed limits on state evolution, the Lieb–Robinson bound

on information propagation in many-body systems, Bell inequalities on correlations achievable by any local hidden-variable theory, and no-cloning or no-broadcasting theorems in quantum information. These results are rarely saturated in realistic settings, yet they are highly informative because they reveal what is fundamentally impossible, rather than what is typically observed.

Taken together, the generality, conceptual clarity, methodological novelty, and potential for direct adaptation to numerical studies and experimental tests underscore the broad significance of our work. We hope that the revised manuscript —and in particular the new Sec. III C— provides clear evidence for both the conceptual importance of our main result and the wider outlook and applications of our techniques, illustrating why it is well suited to a general-interest journal such as Nature Physics.

Final comments

We believe the new version of the manuscript addresses the criticisms of the reviewers and features an improved presentation, both in clarity and in highlighting the significance and impact of our fundamental time-bound for thermalization in quantum mechanics.

Once again, we thank both referees for their attentive assessment of our work.

Yours faithfully,
Paolo Abiuso, Alberto Rolandi, John Calsamiglia, Pavel Sekatski, Martí Perarnau-Llobet.
